# Stability analysis of the incompressible porous media equation and the Stokes transport system via energy structure

**DOI:** 10.1007/s00526-025-03029-y

**Published:** 2025-05-28

**Authors:** Jaemin Park

**Affiliations:** Department of Mathematics, Yonsei University, Seoul, 03722 South Korea

**Keywords:** 76S05, 35Q35, 34D05, 76B03

## Abstract

In this paper, we revisit asymptotic stability for the two-dimensional incompressible porous media equation and the Stokes transport system in a periodic channel. It is well-known that a stratified density, which strictly decreases in the vertical direction, is asymptotically stable under sufficiently small and smooth perturbations. We provide improvements in the regularity assumptions on the perturbation and in the convergence rate. Unlike the standard approach for stability analysis relying on linearized equations, we directly address the nonlinear problem by exploiting the energy structure of each system. While it is widely known that the potential energy is a Lyapunov functional in both systems, our key observation is that the second derivative of the potential energy reveals a (degenerate) coercive structure, which arises from the fact that the solution converges to the minimizer of the energy.

## Introduction

In this paper, we investigate asymptotic stability in the incompressible porous media equation (IPM) and the Stokes transport system in a periodic channel $\Omega :=\mathbb {T}\times (0,1)$. To introduce the models, let us consider a continuity equation with a velocity field *u*(*t*, *x*),

$$ \rho _t +\nabla \cdot (u \rho )=0,\text { in }\Omega \hbox { and }\rho (0,x)=\rho _0(x), $$

for some nonnegative scalar-valued function $\rho _0$, which will be referred to as density throughout the paper. Neglecting physical constants, the velocity *u* in each model is determined by $\rho$ via

$$ {\left\{ \begin{array}{ll} u= -\nabla p -\begin{pmatrix} 0 \\ \rho \end{pmatrix},\text { with } \nabla \cdot u = 0 \text { in }\Omega \text { and } u\cdot \vec {n}=0 \text { on }\partial \Omega , &  \text {(IPM) }\\ \Delta u= -\nabla p -\begin{pmatrix} 0 \\ \rho \end{pmatrix},\text { with } \nabla \cdot u \text { in }\Omega \text { and } u=0 \text { on }\partial \Omega ,&\text {(Stokes) } \end{array}\right. } $$

where $\vec {n}$ denotes the unit normal vector on $\partial \Omega$. We note that, given $\rho$, the above equations (referred to as Darcy’s law and the steady Stokes equation for the IPM and the Stokes transport system, respectively) uniquely determine the velocity *u* and the pressure *p* (up to a constant) (See e.g. [3, Chapter 1] for the IPM and [7, Chapter IV] for the Stokes).

Both the IPM equation and the Stokes transport system describe the evolution of transported density driven by an incompressible fluid subject to gravity. Depending on the physical context, these equations can be interpreted in various ways. However, as our primary focus is on their mathematical analysis, interested readers are directed to [10, 11, 18] for a more detailed exploration of the physical motivations.

In both equations, it is well-known that any stratified density, $\rho _s(x)=\rho _s(x_1,x_2)=\rho _s(x_2)$, which is independent of the horizontal variable $x_1\in \mathbb {T}$, is a steady state. Indeed, the vector field $(0,\rho _s(x_2))^{T}$ can be easily represented as a gradient field. Therefore the velocity determined by either Darcy’s law or the steady Stokes equation corresponds to a trivial vector field $u\equiv 0$. In the paper, our stability analysis will be focused on the stratified densities satisfying the following additional condition:

1.1 $$\begin{aligned} \partial _2\rho _s(x_2)<0,\text { for all }x\in \Omega . \end{aligned}$$

In the remainder of this section, we will provide a brief overview of relevant background information and present our main results separately for each equation.

### The IPM equation

We recall that the IPM equation: For $(t,x)\in \mathbb {R}^+\times \Omega$

1.2 $$\begin{aligned} \text {(IPM)}={\left\{ \begin{array}{ll} \rho _t + u\cdot \nabla \rho = 0,\\ u=-\nabla p - \begin{pmatrix} 0\\ \rho \end{pmatrix},\quad \nabla \cdot u =0, \end{array}\right. } \end{aligned}$$

with boundary condition $u\cdot \vec {n}=0$ on $\partial \Omega$ and $\rho (0,x)=\rho _0(x)$. Thanks to the incompressibility, the velocity can be recovered in terms of the stream function $\Psi$:

1.3 $$\begin{aligned} u=\nabla ^\perp \Psi ,\text { where }\Psi \text { is a solution to } {\left\{ \begin{array}{ll} -\Delta \Psi = \partial _1\rho &  \text { in }\Omega ,\\ \Psi = 0 &  \text { on }\partial \Omega . \end{array}\right. } \end{aligned}$$

We first review well-posedness theory. For strong solutions, the local well-posedness with smooth initial data for the IPM equation (1.1) can be derived by a standard energy estimate in the case where the spatial domain $\Omega$ does not have a boundary. More precisely, when $\Omega =\mathbb {T}^2$ or $\mathbb {R}^2$, any initial data in $H^{k}$ for $k>2$ possesses a unique local-in-time solution $\rho \in C([0,T] ; H^k(\Omega ))$ (see [4, 15]). However, when the boundary is present, the energy estimate becomes more involved. Especially it was proved in [2] that the IPM equation in a periodic channel is locally wellposed in a subspace of $H^k(\Omega )$ for $k\ge 3$ with an additional conditions on the boundary behavior of the solution (See Theorem 3.3 for a more precise statement). Unlike the local well-posedness, the problem of global existence v.s. finite time blow-up for smooth initial data still remains open, while several blow up criteria have been established in [4]. For weak solutions, the global existence of the weak solutions with $L^p$ initial data is currently not available to the best of our knowledge. The nonuniqueness in the class $L^\infty ([0,T]\times \mathbb {T}^2)$ by means of the convex integration [19]. We also note that the global existence of patch-type solutions (the so-called Muskat problem) with the presence of a surface tension have been established in [13] using the optimal transport theory.

While the global well-posedness for general initial data (sufficiently smooth) is still out of reach, there are several stability results near a particular steady state $\rho _s(x_2):=1-x_2$, which, as a byproduct, ensures the global existence. Considering the IPM equation in the spatial domain $\Omega =\mathbb {R}^2$, it was proved in [6] that if $\Vert \rho _0-\rho _s\Vert _{H^k(\mathbb {R}^2)}\le \epsilon$ with $k\ge 20$, the solution converges eventually to $\rho _s$, satisfying $\Vert \rho (t)-\rho _s\Vert _{H^3(\mathbb {R}^2)}\le C\frac{\epsilon }{(1+t)^{1/4}}$. In the same paper, the author also established asymptotic stability in $\Omega =\mathbb {T}^2$ in the class $H^{k}$ for $k\ge 20$ and proved that such perturbed solutions eventually converge to a stratified density, which might not be the same as $\rho _s$. Stability in a periodic channel $\Omega =\mathbb {T}\times (0,1)$ was investigated in [2], proving that in the class $H^k(\Omega )$ for $k\ge 10$ a perturbed solution eventually converges to a stratified density, which again might not be the same as $\rho _s$. While these results requires sufficiently large regularity on the initial perturbation, the authors in [1] recently reduced such regularity assumptions, establishing asymptotic stability in the class $H^k(\mathbb {R}^2)\cap H^{1-s}(\mathbb {R}^2)$ for $k>3$ and $0<s<1$. We also mention the work [14] which proves asymptotic stability in a periodic channel for a perturbation in $H^{k}$ for $3 <k \in \mathbb {N}$, assuming that the vertical derivative of the steady state $\rho _s$ is sufficiently large, depending on the size of the perturbation.

These results concerning asymptotic stability reveal the difficulty to specify a permanent description of the long time limit of the solution. The main challenge stems from the fact that the equilibria of the IPM are not isolated; given two stratified densities $\rho _s(x_2)$ $f(x_2)$, any function of the form $\rho _s(x_2)+\epsilon f(x_2)$ for any $\epsilon >0$ is another equilibrium. However, as noted in [5] the IPM equation is a transport equation with an incompressible flow, therefore as long as such a limit is achieved as a strong limit in $C^1$, each super-level set of the solution $\rho (t)$ must preserve its topological properties and the area. Given an initial data, a stratified density preserving such properties can be uniquely determined as the so-called vertical (decreasing) rearrangement $\rho ^*$,

$$ \rho (x)\mapsto \rho ^*(x):={\frac{1}{2\pi }}\int _0^\infty 1_{\left\{ 0\le x_2 \le |\left\{ \rho > s\right\} |\right\} }ds. $$

Note that such vertical rearrangement is invariant under any measure preserving continuous diffeomorphism, especially, $\rho (t)^*=\rho _0^*$. From this property, one can easily deduce that the long-time limit that was not specified in the above earlier works must be indeed $\rho _0^*$, which can be precisely specified from the initial data.

A somewhat trivial but crucial observation is that $\rho ^*$ is a local minimizer of the potential energy defined as

$$ E_P(\rho ):=\int _{\Omega }\rho (x)x_2dx. $$

More precisely, one can easily deduce that for any measure preserving diffeomorphism $h:\Omega \mapsto \Omega$, it holds that

$$ E_P(\rho ^*\circ h)\ge E_P(\rho ^*), $$

and the equality is obtained if and only if *h* is the identity map. Moreover, the potential energy is a Lyapunov functional to the IPM equation in the sense that given a sufficiently smooth solution $\rho (t)$, namely,

$$ \frac{d}{dt}E_P(\rho (t)) = \int _\Omega u_2\rho dx = \int _{\Omega }u\cdot \begin{pmatrix} 0 \\ \rho \end{pmatrix}dx = -\int _{\Omega }|u|^2 dx, $$

where the last equality is due to Darcy’s law and the incompressibility of the flow. In this view, the solution to the IPM equation can be thought of as a minimizing curve associated to the potential energy whose long-time limit is the ground state of the energy. Hence the asymptotic stability can be obtained by achieving a sufficiently fast decay of $E_P(\rho (t))$ towards $E_P(\rho _0^*)$. Our precise statement of the main theorem for the IPM equation is as follows:

#### [Style2 Style2]Theorem 1.1

Let $k\in \mathbb {N}$ be such that $k\ge 3$ and $\rho _s(x_2):=1-x_2$. There exist $\epsilon =\epsilon (k)>0$ and $C=C(k)>0$ such that if $\rho _0-{\rho }_s\in H^k_0(\Omega )$ and $\Vert \rho _0-{\rho }_s\Vert _{H^k(\Omega )}\le \epsilon$, then there exists a unique solution $\rho \in C([0,\infty ); H^k(\Omega ))$ to the IPM equation (1.2) and it satisfies

$$ \Vert \rho (t)-{\rho }_s\Vert _{H^k(\Omega )}\le C\epsilon ,\text { for all }t>0, $$

Furthermore, the potential energy decays as

$$ E_P(\rho (t))-E_P(\rho _0^*)\le C\epsilon ^2 t^{-k}, $$

where $\rho _0^*$ is the vertical rearrangement of the initial density. Consequently, the solution $\rho (t)$ converges to $\rho _0^*$ satisfying

$$ \Vert \rho (t)-\rho _0^*\Vert _{L^2(\Omega )}\le C \frac{\epsilon }{t^{k/2}},\text { for all }t>0. $$

A few remarks are in order:

#### Remark 1.2

As mentioned earlier, several results concerning asymptotic stability for the IPM equation are available in the literature (e.g., [1, 2, 6]). Compared to these results, our theorem requires slightly weaker regularity on the initial perturbation. Furthermore, our poof in this paper is essentially different from all of these results in that we do not rely on the linearized system but exploit the decay of the potential energy. The key observation of this paper is that the potential energy is not only a Lyapunov functional but also reveals another (degenerate) coercive structure in its second derivative, i.e., $\left( \frac{d}{dt}\right) ^2 E_P(\rho (t))\ge \Vert u_2\Vert _{L^2}^2$. See Subsection 1.3 for more detailed explanation how such property can be used in the proof.

#### Remark 1.3

The author expects that the regularity assumption $H^{k}$ for $k\ge 3$ can be relaxed even further, and a similar strategy used this paper would work out for initial perturbation $\rho _0-\rho _s$ sufficiently small $H^{k}$ for $k> 1+\sqrt{3}$. More concrete evidence of such an expectation will be explained in Subsection 1.3. The main reasons why we do not included such a stronger statement are because we do not want to introduce extra complications of the proof by involving fractional Sobolev spaces, and the local well-posedness of the equation is currently not directly available for lower regularity spaces (see Theorem 3.3). We emphasize that the perturbation regularity cannot be relaxed too much, considering the long time instability result in $H^{2-\epsilon }$ for any $\epsilon >0$ [15, Theorem 1.5]. Noticing that $H^{2}$ barely fails to embed into $C^1$, it seems to be an interesting question whether an asymptotic stability can be established for a small perturbation in $H^{2+\epsilon }$.

#### Remark 1.4

From the stream function formulation (1.3), it is straightforward to see the velocity and the density formally stay in the same regularity class, especially, $\Vert u\Vert _{L^2}\le \Vert \rho -\overline{\rho }\Vert _{L^2}$, where $\overline{\rho }$ is the spatial average of $\rho$. However, compared to the decay rate of the density stated in the above theorem, our argument reveals slightly better decay rates of the kinetic energy and the anisotropic kinetic energy in a time-average sense, that is,

$$ \frac{2}{t}\int _{t/2}^t \Vert u(s)\Vert _{L^2}^2ds \le C_k\frac{\epsilon ^2}{t^{k+1}},\text { and } \frac{2}{t}\int _{t/2}^{t}\Vert u_2(s)\Vert _{L^2}^2 ds\le C_k\frac{\epsilon ^2}{t^{k+2}}, \text { for all }t>0, k\ge 3. $$

#### Remark 1.5

Theorem 1.1 only concerns perturbations near a specific steady state $\rho _s(x_2)=1-x_2$. However, the author expects that our result can be generalized to perturbations near any sufficiently regular stratified density $\rho _s$ such that $\inf _{x\in \Omega }\partial _2\rho _s(x_2)>0$. Again the reason why we do not pursue such a more general statement is due to a lack of an exact statement in the literature about a local well-posedness theorem near general steady states. Instead, we will establish asymptotic stability near general stratified steady states for the Stokes transport system (see Theorem 1.6), which could provide more evident ideas for the IPM equation as well.

### Stokes transport system

The Stokes transport system is another active scalar equation and it shares several interesting properties with the IPM equation. We first recall the system:

1.4 $$\begin{aligned} \text {(Stokes)} = {\left\{ \begin{array}{ll} \rho _t + u\cdot \nabla \rho = 0, & \text {for }(t,x)\in \mathbb {R}^+\times \Omega \\ \nabla \cdot u =0,\\ \Delta u= -\nabla p -\begin{pmatrix} 0 \\ \rho \end{pmatrix}, \quad u=0&\text { on }\partial \Omega . \end{array}\right. } \end{aligned}$$

As in the IPM, the incompressibility condition allows for a stream function formulation for the velocity field:

1.5 $$\begin{aligned} u=\nabla ^\perp \Psi ,\text { where }\Psi \text { solves } {\left\{ \begin{array}{ll} \Delta ^2 \Psi = \partial _1\rho &  \text { in }\Omega ,\\ \Psi =\nabla \Psi =0 &  \text { on }\partial \Omega . \end{array}\right. } \end{aligned}$$

From the stream function formulation, one can easily notice that the velocity in the Stokes transport is much more regular than in the IPM equation. Indeed, such regular structure enables a global well-posedness theorem in a standard manner; if $\rho _0\in H^k(\Omega )$ for $k\ge 3$, then there exists a unique solution $\rho \in C([0,\infty ), H^k(\Omega ))$ (see, [5, Theorem A.1] or [16, Theorem 1.1]). We also mention that the regular of the velocity can yield quite robust structures for weak solutions, for instance, $L^3$ initial data $\rho _0$ yields a unique regular Lagrangian solution for three-dimensional model [12, Theorem 2.2, Theorem 2.4].

Various long time behaviors of the system (1.4) have been investigated in [5], where the authors studied asymptotic stability and boundary layer formation for initial data near $\rho _s(x_2)=1-x_2$. We also mention that the authors in [8, 9] studied the interface problem, where $\rho$ is given as a characteristic function representing two different fluid densities, establishing global existence and asymptotic stability/instability of the interface depending on the Reyleigh-Taylor stability criterion.

In regard to the Stokes transport system, our main result in this paper is a slight extension of the asymptotic stability obtained in [5], especially concerning the regularity assumption on the initial perturbation. This result will be established by adapting a similar strategy that we exploit for the IPM equation based on the energy structure. More precise statement is as follows:

#### [Style2 Style2]Theorem 1.6

Let $\rho _s(x_2)$ be a stratified steady state such that

1.6 $$\begin{aligned} \gamma :=\inf _{x\in \Omega }\left( -\partial _2\rho _s(x_2)\right) >0,\quad \Vert \rho _s\Vert _{H^4(\Omega )} <\infty . \end{aligned}$$

Then there exist $\epsilon =\epsilon (\gamma ,\Vert \rho _s\Vert _{H^4(\Omega )})$ and $C=C(\gamma ,\Vert \rho _s\Vert _{H^4})$ such that if $\rho _0-{\rho }_s\in H^2_0(\Omega )\cap H^4(\Omega )$ and $\Vert \rho _0-{\rho }_s\Vert _{H^4}\le \epsilon$, then the unique solution $\rho \in C([0,\infty ); H^4(\Omega ))$ to the Stokes transport system (1.4) satisfies

$$ \Vert \rho (t)-{\rho }_s\Vert _{H^4(\Omega )}\le C\epsilon ,\text { for all }t>0. $$

Furthermore, the potential energy decays as

$$ E_P(\rho (t))-E_P(\rho _0^*)\le C\epsilon ^2 t^{-2}, $$

where $\rho _0^*$ is the vertical rearrangement of the initial density. Consequently, the solution $\rho (t)$ converges to $\rho _0^*$ satisfying

$$ \Vert \rho (t)-\rho _0^*\Vert _{L^2(\Omega )}\le C\frac{\epsilon }{t},\text { for all }t>0. $$

#### Remark 1.7

A similar asymptotic stability result was already provided in [5, Theorem 1.1], where the authors assumed that the initial perturbation is small in $H^{6}(\Omega )$ near $\rho _s(x_2)=1-x_2$. The authors also provided clear evidence that such a result can be obtained near more general stratified steady state which is sufficiently regular. As noted earlier, the proof presented in this paper is different in that our analysis does not use the linearized equation and it is an energy functional based method. This approach requires a slightly weaker regularity assumption for the initial perturbation. However, as in the IPM equation, there is a threshold of the regularity for the stability. Indeed, [17, Theorem 3.7.2] provides an example of long time instability for small initial perturbation in $H^{2-\epsilon }$ near any steady state.

#### Remark 1.8

As in Remark 1.4, our proof reveals a slight faster decay estimate for the velocity compared to that of the density. More precisely, we obtain

$$ \frac{2}{t}\int _{t/2}^t \Vert \nabla u(s)\Vert _{L^2}^2ds \le C\frac{\epsilon ^2}{t^{3}},\text { and } \frac{2}{t}\int _{t/2}^{t}\Vert u_2(s)\Vert _{L^2}^2 ds\le C\frac{\epsilon ^2}{t^{4}}, \text { for all }t>0. $$

### A sketch of the proof of Theorem 1.1

Let us describe the structure of the proof for the IPM equation. A similar strategy will be adapted to prove the stability for the Stokes transport system.

We consider initial data $\rho _0$ such that $\Vert \rho _0-\overline{\rho }\Vert _{H^k}\le \epsilon$ for sufficiently small $\epsilon >0$ and we will assume $k \ge 3$. For convenience, we denote

$$ \theta (t):= \rho (t)-{\rho }_s,\quad \rho _0^*:=\text { the vertical (decreasing) rearrangement of }\rho _0, $$

and

$$ E(t):=\int _{\Omega } (\rho (t)-\rho _0^*)(x)x_2 dx,\quad K(t):=\Vert \nabla \Psi (t)\Vert _{L^2}^2. $$

Thanks to the weight, $x_2$, in the integral expression for *E*, it is evident that $\rho _0^*$ is the unique minimizer of $\rho \mapsto E_P(\rho )$ among all the functions which can be obtained by a pushforward of $\rho _0$ under a measure preserving map. Indeed, one can adapt a proof similar to that of the Hardy-Littlewood inequality to rigorously justify this fact. Also if $\rho _0^*$ is a non-degenerate minimizer, then $\rho \mapsto E_P(\rho )$ is expected to satisfy a quadratic lower bound in a suitable space. In this paper, we will look for such a lower bound in $L^2(\Omega )$ and establish in Proposition 2.5 that

1.7 $$\begin{aligned} E(t)\sim \Vert \rho (t)- \rho _0^*\Vert _{L^2}^2. \end{aligned}$$

Furthermore, as long as the solution $\rho (t)$ stays close to a stratified density in the space $H^3(\Omega )$, the fact that $H^3(\Omega )$ continuously embeds into $C^1(\Omega )$ suggests that each level set of $\rho (t)$ is also a graph of the horizontal variable $x_1$, from which one can infer that

1.8 $$\begin{aligned} \Vert \rho (t)-\rho _0^*\Vert _{L^2}\le C\Vert \partial _1\rho (t)\Vert _{L^2}. \end{aligned}$$

On the other hand, the time derivative of *E*(*t*) can be computed as

1.9 $$\begin{aligned} \frac{d}{dt}E(t)&=\frac{d}{dt}\int \rho (t,x)x_2dx = \int u_2 \rho dx = \int \partial _1\Psi \rho dx \nonumber \\&= -\int \Psi \partial _1 \rho dx = \int \Psi \Delta \Psi dx = - K(t). \end{aligned}$$

The Biot-Savart law in (1.3) and the Gargliado-Nirenberg inequality tell us that

$$ \Vert \partial _1\rho \Vert _{L^2} =\Vert \Delta \Psi \Vert _{L^2}\le C_k \Vert \nabla \Psi \Vert _{H^k(\Omega )}^{1/k}\Vert \nabla \Psi \Vert _{L^2}^{(k-1)/k}. $$

Combining this with (1.7) and (1.8), we get

$$ K(t)=\Vert \nabla \Psi \Vert _{L^2}^2 \ge _C \Vert \partial _1\rho \Vert _{L^2}^{2k/(k-1)}\Vert \nabla \Psi \Vert _{H^k}^{-{2/(k-1)}}\ge _C E(t)^{k/(k-1)}\Vert \nabla \Psi \Vert _{H^k}^{-2/(k-1)}. $$

Substituting this into (1.9), we obtain

1.10 $$\begin{aligned} \frac{d}{dt}E(t) \le _C -E(t)^{k/(k-1)}\Vert \nabla \Psi (t)\Vert _{H^k}^{-2/(k-1)}. \end{aligned}$$

This inequality is the main source of the asymptotic stability. Let us use the following notation which is slightly different from the usual convention: For $\alpha >0$ and $f:\mathbb {R}^+\mapsto \mathbb {R}^+$,

1.11 $$\begin{aligned} f(t) = O(t^{-\alpha }),\text { if }\frac{2}{t}\int _{t/2}^t f(s)ds\le C(1+t)^{-\alpha }\text { for some }C>0. \end{aligned}$$

Clearly $f(t)=O(t^{-\alpha })$ means that *f* decays like $t^{-\alpha }$ in average. Let us make an ansatz:

1.12 $$\begin{aligned} \Vert \theta (t)\Vert _{H^k}\lesssim \epsilon ,\quad \Vert \nabla \Psi (t)\Vert _{H^k} = O(t^{-\alpha }), \text { for all }t>0, \text {for some }\alpha >0 \end{aligned}$$

Under this ansatz, one can deduce from the inequality (1.10) that

1.13 $$\begin{aligned} E(t)= O({t^{-(k+2\alpha -1)}}). \end{aligned}$$

This estimate will be rigorously proved in Corollary 3.8, using the lemmas in Subsection 2.2. With this energy decay rate, the energy variation in time (1.9) suggests that *K*(*t*) decays faster than *E*(*t*) by a factor of $t^{-1}$. Indeed, this elementary heuristic can be made rigorous by measuring the decay rates as an average (Lemma 2.2). Hence we can deduce

$$ K(t) = O(t^{-(k+2\alpha )}). $$

Having such a decay rate for *K*(*t*), we will proceed to look at a higher derivative of the potential energy in time. A key observation is that the second derivative of the energy *E*(*t*) also exhibits a coercive structure, namely,

$$ \left( \frac{d}{dt}\right) ^2 E(t) = -\frac{d}{dt}K(t) \ge C \Vert u_2(t)\Vert _{L^2}^2, $$

which is the result of Proposition 3.7. We emphasize that such coercive structure should not come as a surprise, because the solution is expected to converge to a non-degenerate minimizer of the potential energy. Again, our notation (1.11) allows us to postulate that $\Vert u_2\Vert _{L^2}^2$ will decay faster than *K*(*t*) by a factor $t^{-1}$, that is,

1.14 $$\begin{aligned} \Vert u_2(t)\Vert _{L^2}^2 = O(t^{-(k+2\alpha +1)}). \end{aligned}$$

So far, the decay rates of the energies have been derived under the ansatz (1.12), therefore it must be justified in order to close the argument. To this end, in Proposition 3.6, we will derive the following estimate (an informal estimate is presented at this point for simplicity):

1.15 $$\begin{aligned} \frac{d}{dt}\Vert \theta (t)\Vert _{H^k}^2 + \Vert \nabla \Psi (t)\Vert _{H^k}^{2}\le C \Vert u_2\Vert _{W^{1,\infty }}\Vert \theta (t)\Vert _{H^k}^2. \end{aligned}$$

Using again the Gagliardo-Nirenberg interpolation inequality and Young’s inequality, we deduce

$$\begin{aligned} \Vert u_2\Vert _{W^{1,\infty }}&\le C\Vert u_2\Vert _{L^2}^{1-2/k}\Vert u_2\Vert _{H^k}^{2/k}\le C\Vert u_2\Vert _{L^2}^{1-2/k}\Vert \nabla \Psi \Vert _{H^k}^{2/k}\le \eta \Vert \nabla \Psi \Vert _{H^k}^2 \\&\quad + C_\eta \Vert u_2\Vert _{L^2}^{(k-2)/(k-1)}, \end{aligned}$$

for any $\eta \ll 1$. Noting that $\Vert \theta (t)\Vert _{H^k}\lesssim \epsilon \ll 1$ as long as the ansatz (1.12) is valid, we substitute this estimate into (1.15), yielding that

1.16 $$\begin{aligned} \frac{d}{dt}\Vert \theta (t)\Vert _{H^k}^2 + \Vert \nabla \Psi (t)\Vert _{H^k}^{2}\le C\Vert u_2\Vert _{L^2}^{(k-2)/({k-1})}\Vert \theta (t)\Vert _{H^k}^2= \epsilon ^2O( t^{-(k+2\alpha +1)(k-2)/(2k-2)}), \end{aligned}$$

where the last equality is due to (1.14). In this differential inequality, a sufficient condition for the ansatz (1.12) to persist is that the right-hand side should decay faster than $O(t^{-1})$, that is,

1.17 $$\begin{aligned} \frac{(k+2\alpha +1)(k-2)}{2k-2} > 1. \end{aligned}$$

Indeed, if this condition is satisfied, integrating the both sides of (1.16) in time yields that

$$ \sup _{t>0}\Vert \theta (t)\Vert _{H^k}^2 + \int _0^{\infty }\Vert \nabla \Psi (t)\Vert _{H^k}^2dt \le C_{k,\alpha ,\theta _0} \epsilon ^2. $$

In this case, $t\mapsto \Vert \nabla \Psi (t)\Vert _{H^k}^2$ is integrable in time, which indicates that our ansatz (1.12) should hold at least for some $\alpha \ge 1/2$;

$$ \frac{2}{t}\int _{t/2}^{t}\Vert \nabla \Psi (s)\Vert _{H^k}ds\le \left( \frac{2}{t}\int _{t/2}^t \Vert \nabla \Psi (s)\Vert _{H^k}^2ds\right) ^{1/2}\le C_{k,\alpha ,\theta _0}\epsilon \sqrt{\frac{2}{t}} \text { for all } t>0. $$

For $\alpha \ge 1/2$, the minimum value of *k* for the sufficient condition (1.17) to hold can be directly computed:

$$ \frac{(k+2\alpha +1)(k-2)}{2k-2}>1 \Longleftarrow \frac{(k+2)(k-2)}{2k-2}> 1\Longleftarrow k> 1+\sqrt{3}. $$

The range of *k* stated in Theorem 1.1 is strong enough to satisfy the sufficient condition for the above scheme. Especially, (1.13) with $\alpha \ge 1/2$ directly gives the decay rate of the potential energy stated in the Theorem 1.1, resulting in the desired asymptotic stability.

### Organization of the paper

In Section 2, we collect useful tools concerning simple ODE problems and quantitative estimates for the potential energy. The stability analysis for the IPM equation and the Stokes transport system will be separately investigated in Section 3 and Section 4 respectively.

## Preparation: Time-average decay and vertical rearrangement

### Conventional notations

Following the conventional practice, we denote by *C* an implicit positive constant that may vary from line to line. In the case where *C* depends on a quantity, say *A*, we will represent it as $C_A$ or *C*(*A*). For two quantities, *A* and *B*, we will also use the notation $A \le _C B$, indicating that $A \le CB$ for some constant $C > 0$. We denote

2.1 $$\begin{aligned} C^\infty _0(\Omega ):=\left\{ f\in C^\infty (\Omega ): {\text {supp}(f)}\subset \Omega \right\} , \end{aligned}$$

where $\text {supp}(f)$ is the closed support of *f*.

### Time-average decay rates in differential inequalities

In the proof of asymptotic stability, we will measure the decay rates of the energy quantities in a time-average manner. To prepare for this analysis, we will gather useful lemmas concerning simple differential inequalities. In what follows [0, *T*] will denote an arbitrary time interval for some $T>0$.

#### Lemma 2.1

Let $\alpha >0,\ 1<n$. Let *a*(*t*) and *f*(*t*) be nonnegative functions on [0, *T*] such that

$$ \frac{d}{dt}f(t) \le - a(t)^{-\alpha }f(t)^n,\quad f(0)=f_0. $$

Then, *f* satisfies

$$ f(t)\le _{\alpha ,n} \frac{A^{\alpha /(n-1)}}{t^{(\alpha +1)/(n-1)}},\text {for all }t\in [0,T],\text { where }A:=\int _0^t a(s)ds. $$

#### Proof

Dividing the differential inequality by $f(t)^n$ and integrating it in time, we find that

2.2 $$\begin{aligned} \frac{1}{f^{n-1}(t)}-\frac{1}{f_0^{n-1}} \ge _{n} \int _0^t a(s)^{-\alpha }ds. \end{aligned}$$

Since $\alpha >0$, Jensen’s inequality yields that $\int _0^t a(s)^{-\alpha }\frac{ds}{t} \ge \left( \int _0^t a(s)ds\right) ^{-\alpha }t^\alpha = A^{-\alpha }t^{\alpha },$ which implies

$$ \int _0^t a(s)^{-\alpha }ds \ge _n A^{-\alpha }t^{\alpha +1}. $$

Plugging this into (2.2), we get $\frac{1}{f^{n-1}(t)}\ge _n A^{-\alpha }t^{\alpha +1}$, which immediately gives the desired result.

#### Lemma 2.2

Let $n>0$ and *f*(*t*), *g*(*t*), *h*(*t*) be nonnegative functions on [0, *T*] such that

2.3 $$\begin{aligned} \frac{d}{dt}f(t)\le -g(t),\ \frac{d}{dt}g(t)\le -h(t),\text { and } f(t)\le \frac{C}{t^n}, \end{aligned}$$

for some $C>0$. Then, it holds that

$$ \frac{2}{t}\int _{t/2}^t g(s)ds\le _n \frac{C}{t^{n+1}},\text { and } \frac{2}{t}\int _{t/2}^{t}h(s)ds\le _n \frac{C}{t^{n+2}},\text { for all }t\in [0,T]. $$

#### Proof

Let us choose $s,t\in [0,T]$ arbitrary so that $0\le s\le t\le T$. Integrating $f'(t)\le -g(t)$ over [*s*, *t*] for $s\in (0,t)$, we get $f(t)-f(s)+\int _s^t g(u)du\le 0$. Hence, the upper bound of *f*(*t*) tells us that

2.4 $$\begin{aligned} \int _s^t g(u)du \le f(s)\le \frac{C}{s^{n}},\text { for }0<s<t<T. \end{aligned}$$

Plugging in $s=t/2$, we see that

2.5 $$\begin{aligned} \frac{2}{t}\int _{t/2}^t g(u)du\le \frac{C}{t^{n+1}}, \end{aligned}$$

and this is the desired estimate for *g*.

Similarly, we integrate $g'\le -h$ and observe that

2.6 $$\begin{aligned} \int _{s}^t h(u)du \le g(s),\text { for }0<s<t<T. \end{aligned}$$

Integrating one more time in *s* over [*t*/4, *t*], we find that the left-hand side must satisfy

$$\begin{aligned} \int _{t/4}^t \int _s^th(u)duds&= \int _{t/4}^th(u)\int _{t/4}^udsdu =\int _{t/4}^t h(u)(u-t/4)du \\&\ge \int _{t/2}^t h(u)(u-t/4)du \ge \frac{t}{4}\int _{t/2}^th(u)du. \end{aligned}$$

On the other hand, integrating the right-hand side in (2.6) over [*t*/4.*t*] yields

$$ \int _{t/4}^t g(s)ds = \int _{t/4}^{t/2}g(s)ds + \int _{t/2}^t g(s)ds\le _n \frac{C}{(t/2)^{n}}+\frac{C}{t^{n}}\le _n \frac{C}{t^{n}}, $$

where we used (2.5). Putting them together, we obtain

$$ t\int _{t/2}^{t}h(u)du\le _n \frac{C}{t^{n}}, $$

Dividing the both sides by $t^2$, we derive the desired estimate for *h*, finishing the proof.

It is an elementary fact that if a bounded function *f* exhibits a decay rate $O(t^{-(1+\epsilon )})$, it is integrable over all time, i.e., $\int _0^\infty f(t) dt<C_\epsilon <\infty$. In the next lemma, we demonstrate that if *f*(*t*) decays like $O(t^{1+\epsilon })$ in a time-average sense, the same conclusion holds.

#### Lemma 2.3

Let $T> 2$ and $n> 1$. Let *f*(*t*) be a nonnegative function on [0, *T*] such that

$$ \frac{2}{t}\int _{t/2}^{t} f(s)ds\le \frac{E}{t^n},\text { for some } E>0,\text { for all }t\in [2,T]. $$

Then, for $\alpha \in (1/n,1]$, we have

$$ \int _1^T f(t)^{\alpha }ds \le C_{\alpha ,n}E^{\alpha }, $$

where $C_{\alpha ,n}>0$ does not depend on *T*.

#### Proof

We pick $N\in \mathbb {N}$ such that

2.7 $$\begin{aligned} \frac{T}{2^{N+1}}\le 1\le \frac{T}{2^{N}}\le 2, \end{aligned}$$

and define $T_{i}:={2^{-i}}T$, for $i=0,\ldots , N$. We decompose

$$ \int _1^T f(t)^{\alpha }ds = \int _{1}^{T_N}f(t)^\alpha dt + \sum _{i=1}^{N}\int _{T_i}^{T_{i-1}} f(t)^\alpha dt. $$

Since $T_N\le 2$ and $\alpha \le 1$, applying Jensen’s inequality, we obtain

2.8 $$\begin{aligned} \int _{1}^{T_N}f(t)^\alpha dt\le \int _1^2 f(t)^\alpha dt \le \left( \int _1^2 f(t)dt \right) ^\alpha \le E^\alpha . \end{aligned}$$

For $1\le i\le N$, again Jensen’s inequality gives us that

$$\begin{aligned} \int _{T_i}^{T_{i-1}} f(t)^\alpha dt&\le \left( \frac{1}{T_{i-1}-T_{i}}\int _{T_i}^{T_{i-1}}f(t)dt \right) ^\alpha |T_{i-1}-T_i| \\&=\left( \frac{1}{T_i}\int _{T_i}^{T_{i-1}}f(t)dt\right) ^{\alpha }T_i\le _{\alpha } E^\alpha T_i^{1-\alpha n}, \end{aligned}$$

where the last inequality follows from the decay hypothesis for *f*. Summing over $i=1,\ldots ,N$, we get

$$ \sum _{i=1}^N\int _{T_i}^{T_{i-1}} f(t)^\alpha dt\le _\alpha E^\alpha T^{1-\alpha n }\sum _{i=1}^{N}\left( {2^{\alpha n-1}}\right) ^{i}\le _{\alpha ,n} E^\alpha \left( \frac{T}{2^N}\right) ^{1-\alpha n}\le C_{\alpha ,n}E^\alpha , $$

where the last inequality follows from (2.7). Combining this with (2.8), we finish the proof.

### Vertical rearrangement

Given a Borel measurable function *f* on $\Omega$, we define its vertical (decreasing) rearrangement as

2.9 $$\begin{aligned} f^*(x_2):=\int _0^\infty 1_{\left\{ 0\le x_2 \le |\left\{ f > s\right\} |\right\} }ds. \end{aligned}$$

By its definition, it is clear that $x_2\mapsto f^*(x_2)$ is monotone decreasing. In the rest of the section, we consider a stratified density $\rho _s(x)=\rho _s(x_2)$, a function *f* that is close to $\rho _s$ in a Sobolev space and its vertical rearrangement.

Before presenting the lemmas, let us collect some basic properties for $\rho _s$. We will always assume that

2.10 $$\begin{aligned} \gamma :=\inf _{\Omega }(-\partial _2\rho (x_2))>0,\quad \Vert \rho _s\Vert _{H^4(\Omega )} < \infty . \end{aligned}$$

By the monotonicity of $\rho _s$, we can describe the image of $\rho _s$ as

2.11 $$\begin{aligned} I:=\rho _s(\Omega )= [\rho _s(1),\rho _s(0)]. \end{aligned}$$

The inverse function theorem, combined with the assumption that $\gamma >0$, guarantees the existence of the inverse of $\rho _s$, that is, $\phi _0:=\rho _s^{-1}:I\mapsto [0,1]$ is well-defined. Moreover, since $\rho _s$ depends on the single variable $x_2$, the regularity assumption in (2.10), combined with the usual Sobolev embedding theorem, ensures that $\rho _s\in C^3(\Omega )$, and $\Vert \rho _s\Vert _{C^3}\le _C \Vert \rho _s\Vert _{H^4}.$ With such information, one can straightforwardly deduce the following estimates:

2.12 $$\begin{aligned} \Vert \partial _s\phi _0\Vert _{L^\infty }+\Vert \partial _{ss}\phi _0\Vert _{L^\infty } + \Vert \partial _2\rho _s\Vert _{L^\infty }+\Vert \partial _{22}\rho _s\Vert _{L^\infty }+\Vert \partial _{222}\rho _s\Vert _{L^{\infty }}\le C(\gamma ,\Vert \rho _s\Vert _{H^4}). \end{aligned}$$

Noting that $H^3(\Omega )$ continuously embeds into $C^1(\Omega )$, one can infer that if a function *f* is sufficiently close to $\rho _s$ in $H^3$, similar properties of the level sets and the inverse function of *f* can be quantitatively estimated. This is will be the main implication of the next lemma. In the rest of this section, the implicit constant *C*, that appears in the proofs, may depend on $\gamma$ and $\Vert \rho _s\Vert _{H^4}$ but we will omit its dependence in the notation for simplicity.

#### Lemma 2.4

Suppose $\rho _s$ satisfies (2.10). There exists $0<\delta =\delta (\gamma ,\Vert \rho _s\Vert _{H^4})\ll 1$, such that if

$$ f={\rho }_s\text { on }\partial \Omega \text { and } \Vert f-{\rho }_s\Vert _{H^3(\Omega )}\le \delta , $$

then there exist $\phi _1:I\mapsto [0,1]$ and $h:\mathbb {T}\times I\rightarrow [0,1]$ such that

$$\begin{aligned} \int _{\mathbb {T}}h(x_1,s)dx_1=0\text { and } f(x_1,\phi _1(s)+h(x_1,s))= s = f^*(\phi _1(s)),\text { for }(x_1,s)\in \mathbb {T}\times I. \end{aligned}$$

Furthermore, the following estimates hold:

2.13 $$\begin{aligned} \Vert \partial _{ss}(\phi _1-\phi _0)\Vert _{L^\infty }+ \Vert \partial _s(\phi _1-\phi _0)\Vert _{L^\infty } + \Vert h\Vert _{L^\infty }+\Vert \partial _sh\Vert _{L^\infty }\le C \Vert f-{\rho }_s\Vert _{H^3}. \end{aligned}$$

where $C>0$ is a constant which depends on $\Vert \rho _s\Vert _{H^4}$ and $\gamma$.

**Fig. 1:**
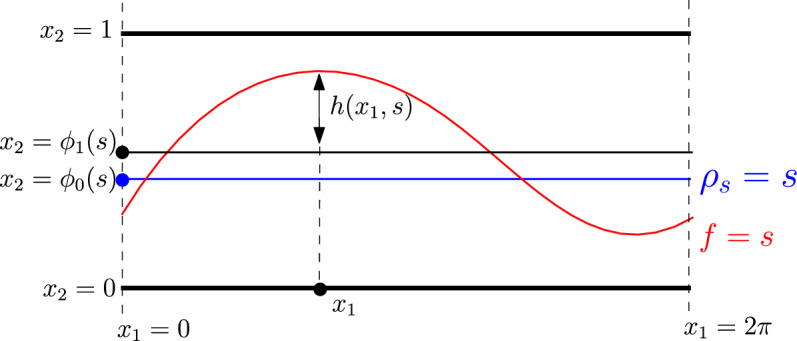
For each $s\in I$, $\phi _1$ is uniquely determined so that the deviation $x_1\mapsto h(x_1,s)$ has zero average in $x_1$

An illustration for the lemma is presented in Figure 1.

#### Proof

We notice that the image of *f* is exactly equal to the interval *I*, which is the image of ${\rho }_s$. Indeed, we have

2.14 $$\begin{aligned} \partial _2 f (x_1,x_2)&= \partial _2{\rho }_s(x_2) + (\partial _2 f(x_2) - \partial _2 {\rho }_s(x_2))\le -\gamma + C\Vert f-{\rho }_s\Vert _{H^3}\le -\gamma +C\delta < 0, \end{aligned}$$

for sufficiently small $\delta >0$. Hence, $x_2\mapsto f(x_1,x_2)$ is strictly decreasing. Since $f={\rho }_s$ on $\partial \Omega$, it follows that $f(\overline{\Omega })=I$.

Thanks to the monotonicity, the implicit function theorem tells us that there is a parametrization of the level curves, $\left\{ f=s\right\}$, which we will denote by $\phi (\cdot ,s)$, that is,

2.15 $$\begin{aligned} f(x_1,\phi (x_1,s))=s\text { for each }(x_1,s)\in \mathbb {T}\times I. \end{aligned}$$

Let us rewrite it as

2.16 $$\begin{aligned} \phi (x_1,s) = \phi _0(s) + g(x_1,s), \text { where } g(x_1,s):=\phi (x_1,s) -\phi _0(s). \end{aligned}$$

Now, we will aim to derive necessary estimates for *g*. Writing

$$\begin{aligned} 0 = \rho _s(\phi _0(s))-f(x_1,\phi (s)) = \rho _s(\phi _0(s)) - \rho _s(\phi (x_1,s))+ \left( \rho _s(\phi (x_1,s)) -f(x_1,\phi (x_1,s)) \right) , \end{aligned}$$

we notice that

2.17 $$\begin{aligned} \int _{0}^{g(x_1,s)}\partial _2\rho _s(y+\phi _0(s))dy= \rho _s(\phi (x_1,s)) -f(x_1,\phi (x_1,s)). \end{aligned}$$

Since $\partial _2\rho _s<-\gamma <0$ and $\Vert f-\rho _s\Vert _{L^\infty }\le _C \Vert f-\rho _s\Vert _{H^3}$, we find that

2.18 $$\begin{aligned} \Vert g\Vert _{L^\infty }\le _C \Vert f-\rho _s\Vert _{H^3}. \end{aligned}$$

Differentiating (2.17) with respect to *s*, we obtain

2.19 $$\begin{aligned} \partial _s g(x_1,s)\partial _2\rho _s (\phi (x_1,s))&+ \int _{0}^{g(x_1,s)}\partial _{22}\rho _s(y+\phi _0(s))\partial _s\phi _0(s)dy \nonumber \\&= (\partial _2\rho _s - \partial _2f)(x_1,\phi (x_1,s))\partial _s\phi (x_1,s). \end{aligned}$$

Again, using $\partial _2\rho _s<-\gamma <0$, we have $|\partial _s g\partial _2\rho _s |\ge C | \partial _sg|$. On the other hand, using (2.12) and (2.18), we can estimate the integral on the left-hand side as

$$\begin{aligned} \left| \int _{0}^{g(x_1,s)}\partial _{22}\rho _s(y+\phi _0(s))\partial _s\phi _0(s)dy\right| \le _C \Vert f-\rho _s\Vert _{H^3}. \end{aligned}$$

For the right-hand side, at each point $(x_1,s)\in \mathbb {T}\times I$, we have

$$\begin{aligned} |(\partial _2\rho _s - \partial _2f)\partial _s\phi |\le _C \Vert \rho _s-f\Vert _{H^3}|\partial _s\phi | \le _C \Vert \rho _s-f\Vert _{H^3} |\partial _s\phi _0| + \delta |\partial _s g|\le _C \Vert \rho _s-f\Vert _{H^3} + \delta |\partial _s g|. \end{aligned}$$

Assuming $\delta$ is sufficiently small depending on $\gamma$, these estimates and (2.19) give us

2.20 $$\begin{aligned} \Vert \partial _s g\Vert _{L^\infty }\le _C \Vert f-\rho _s\Vert _{H^3}. \end{aligned}$$

Next let us estimate $\partial _{ss}g$. Once again, differentiating (2.19) in *s* and using the chain rule, we get (at each point $(x_1,s)$)

2.21 $$\begin{aligned} \partial _{ss}g \partial _2 \rho _s(\phi )&=A_1+A_2+A_3+A_4+A_5+A_6, \end{aligned}$$

where

$$\begin{aligned} A_1&:=-\partial _s g \partial _{22}\rho _s(\phi )\partial _s\phi ,\\ A_2&:=-\partial _sg\partial _{22}\rho _s(\phi )\partial _s\phi _0\\ A_3&:=-\int _{0}^{g}\partial _{222}\rho _s(y+\phi _0)(\partial _s\phi _0)^2dy \\ A_4&:=-\int _0^{g}\partial _{22}\rho _s(y+\phi _0)\partial _{ss}\phi _0(s)dy,\\ A_5&:=\partial _{22}(\rho _s-f)(\phi )(\partial _s\phi )^2\\ A_6&:=\partial _{2}(\rho _s-f)(\phi ) \partial _{ss}\phi . \end{aligned}$$

Again, $\partial _2\rho _s\le -\gamma <0$ gives us that the left-hand side of (2.21) can be estimated as

$$ |\partial _{ss}g\partial _2\rho _s(\phi )|\ge C |\partial _{ss}g|. $$

Using (2.12), (2.18) and (2.20), it is straightforward that

$$\begin{aligned}&|A_1|, |A_2|, |A_3|, |A_4| \le _C \Vert f-\rho _s\Vert _{H^3},\quad |A_5|\le _C |\partial _{22}(\rho _s-f)|, \\  &\quad |A_6|\le _C \Vert f-\rho _s\Vert _{H^3} + \delta | \partial _{ss}g|. \end{aligned}$$

Plugging them into (2.21), we obtain a pointwise estimate:

2.22 $$\begin{aligned} |\partial _{ss}g(x_1,s)|&\le _C \Vert f-\rho _s\Vert _{H^3} + |\partial _{22}\rho _s(\phi (x_1,s))-\partial _{22}f(x_1,\phi (x_1,s))|. \end{aligned}$$

Then for each fixed $x_1$, we treat the functions above as a function of *s*. Applying the Sobolev embedding $W^{1,1}(I)\mapsto L^\infty (I)$, we have that for each $x_1\in \mathbb {T}$,

2.23 $$\begin{aligned} \sup _{s\in I}&|\partial _{22}\rho _s(\phi (s)) - \partial _{22}f(\phi (s))|\nonumber \\&\le _C \int _{I} |\partial _s(\partial _{22}(\rho _s-f)(\phi (s)))|ds + \int _{I}|\partial _{22}(\rho _s-f)(\phi (s))|ds\nonumber \\&\le _C \int _{I}|\partial _{222}(\rho _s-f)(\phi (s))\partial _s\phi (s)|ds + \int _{I}|\partial _{22}(\rho _s-f)(\phi (s))|ds \nonumber \\&\le _C\int _{0}^{1}|\partial _{222}(\rho _s-f)(x_1,x_2)|dx_2 + \int _{0}^{1}|\partial _{22}(\rho _s- f)(x_1,x_2)|dx_2\left( \sup _{s}|\partial _s\phi (s)|^{-1}\right) , \end{aligned}$$

where we used the change of variables $\phi (s)\rightarrow x_2$ in the last inequality. Note that

$$ |\partial _s\phi (s)| \ge |\partial _s\phi _0(s)| - |\partial _s g(s)|\ge \gamma - \delta >C, $$

where the second inequality is due to (2.20) and the last inequality follows from the assumption that $\delta$ is sufficiently small compared to $\gamma$. Hence, integrating (2.23) in $x_1$ over $\mathbb {T}$, we get

$$\begin{aligned} \int _{\mathbb {T}} \sup _{s\in I} |\partial _{22}\rho _s(\phi (s)) - \partial _{22}f(\phi (s))|dx_1\le _C \Vert \rho _s-f\Vert _{W^{3,1}(\Omega )} + \Vert \rho _s-f\Vert _{W^{2,1}(\Omega )} \le _C \Vert \rho _s-f\Vert _{H^3(\Omega )}. \end{aligned}$$

Combining this with (2.22), we arrive at

2.24 $$\begin{aligned} \sup _{s\in I}\int _{\mathbb {T}}|\partial _{ss}g(x,s)|dx \nonumber \\&\le \Vert f-\overline{\rho }\Vert _{H^3(\Omega )}. \end{aligned}$$

Towards the proof, we define $\phi _1$ and *h* as follows:

$$ \phi _1 := \phi _0(s) + \frac{1}{2\pi }\int _{\mathbb {T}}g(x,s)dx,\quad h(x,s):=g(x,s)-\int _{\mathbb {T}}g(x,s)dx. $$

Let us check if $\phi _1$ and *h* satisfy the desired properties. By its definition, we have $\int _{\mathbb {T}}h(x,s)dx=0$ for each $s\in I$. Also, (2.15) and (2.16) tells us that

$$ f(x,\phi _1(s)+h(x,s)) = f(x,\phi _0(s)+g(x,s)) =f (x,\phi (x,s)) = s. $$

Collecting the estimates for *g* obtained in (2.18), (2.20) and (2.24), we see that

2.25 $$\begin{aligned} \Vert h\Vert _{L^\infty }, \Vert \partial _s h\Vert _{L^{\infty }(\mathbb {T}\times I)},\ \Vert \partial _s(\phi _1-\phi _0)\Vert _{L^{\infty }(\mathbb {T}\times I)}, \ \Vert \partial _{ss}(\phi _1-\phi _0)\Vert _{L^{\infty }(\mathbb {T}\times I)}\le C\Vert f-\overline{\rho }\Vert _{H^3(\Omega )}, \end{aligned}$$

which proves (2.13). To finish the proof, we have to prove $f^*(\phi _1(s)) = s.$ To this end, observe that for each $s\in I$, it holds that

$$ \left| \left\{ (x_1,x_2)\in \Omega : \phi _1^{-1}> s\right\} \right| = \int _{\mathbb {T}}\int _{0}^11_{\left\{ \phi _1^{-1}(x_2)\ge s\right\} }dx = \int _{\mathbb {T}}\int _0^{\phi _1(s)}dx = 2\pi \phi _1(s). $$

We also have

$$\begin{aligned} \left| \left\{ (x_1,x_2)\in \Omega : {f}> s\right\} \right|&= \int _{\mathbb {T}}\int _{0}^1 1_{\left\{ f(x_1,x_2)>s\right\} }dx =\int _{\mathbb {T}}\int _0^11_{\left\{ x_2\le \phi (x_1,s)\right\} }dx\\&=\int _\mathbb {T}\phi (x_1,s)dx_1 = 2\pi \left( \phi _0(s)+\frac{1}{2\pi }\int _{\mathbb {T}}g(x_1,s)dx_1\right) \\&= 2\pi \phi _1(s). \end{aligned}$$

This implies that the areas of every super-level set of $\phi _1^{-1}$ and *f* are equal. Recalling the definition of the rearrangement in (2.9), we arrive at

$$ f^*(x_2) = \int _0^\infty 1_{0\le x_2\le |\left\{ f> s\right\} |} ds =\int _0^\infty 1_{0\le x_2\le |\left\{ \phi _1^{-1}> s\right\} |} ds = \phi ^{-1}(x_2). $$

This proves $$f^*(\phi _1(s)) = s.$$

Recall that for $f\in L^1(\Omega )$, its potential energy is defined as

2.26 $$\begin{aligned} E_P(f):=\int _\Omega f(x)x_2 dx. \end{aligned}$$

It is well-known that for any stratified function $\rho _s$ is a critical point of $E_P$ under any divergence-free perturbation. In the case where *f* is sufficiently close to $\rho _s$, the next proposition will quantitatively demonstrate that $f^*$ is the unique minimizer of $E_P$ among all functions with the same area of super-level sets.

#### Proposition 2.5

Suppose $\rho _s$ satisfies (2.10). There exists $\delta =\delta (\gamma ,\Vert \rho _s\Vert _{H^4})>0$ such that if $f={\rho }_s$ on $\partial \Omega$ and $\Vert f-{\rho }_s\Vert _{H^3(\Omega )}\le \delta$, then

2.27 $$\begin{aligned} C^{-1} \Vert f-f^*\Vert _{L^2(\Omega )}^2\le E_P(f)-E_P(f^*) \le C\Vert f-f^*\Vert _{L^2(\Omega )}^2. \end{aligned}$$

Moreover, we have

2.28 $$\begin{aligned} \Vert \partial _1f \Vert _{L^2(\Omega )}\ge C\Vert f-f^*\Vert _{L^2(\Omega )}. \end{aligned}$$

The constant *C* depends only on $\gamma$ and $\Vert \rho _s\Vert _{H^4}$.

#### Proof

Let us prove (2.27) first. For sufficiently small $\delta >0$, Lemma 2.4 ensures the existence of $\phi _1,h$ such that

2.29 $$\begin{aligned} \ \int _{\mathbb {T}}h(x,s)dx&= 0,\quad f(x,\phi _1(s)+h(x,s))=s=f^*(\phi _1(s)), \end{aligned}$$

and

2.30 $$\begin{aligned} \Vert \partial _{ss}(\phi _1-\phi _0) \Vert _{L^\infty }+ \Vert \partial _s\phi _1 -\partial _s\phi _0\Vert _{L^\infty } + \Vert h\Vert _{L^\infty }+\Vert \partial _sh\Vert _{L^\infty }\le C\delta , \end{aligned}$$

where $\phi _0$ is the inverse of $\rho _s$, which trivially implies $\partial _s\phi _0(s)=\frac{1}{\partial _2\rho _s(\phi _0(s))}$. From (2.10), it follows that

2.31 $$\begin{aligned} 0< \frac{1}{C}\le -\partial _s\phi _0 <C. \end{aligned}$$

Since $f^*$ is the inverse of $\phi _1$, the inverse function theorem and the estimates for $\phi _1$ in (2.30) tell us that

2.32 $$\begin{aligned} \Vert \partial _{22}f^*-\partial _{22}\rho _s\Vert _{L^\infty (\Omega )}+ \Vert \partial _{2}f^*-\partial _2\rho _s\Vert _{L^\infty (\Omega )}\le C\delta . \end{aligned}$$

Denoting $\phi (x,s):=\phi _1(s)+h(x_1,s)$ and using the change of variables, $x_2\rightarrow \phi (x_1,s)$, we have

2.33 $$\begin{aligned} \Vert f-f^*\Vert _{L^2(\Omega )}^2&= \int _{\mathbb {T}}\int _I |f(x,\phi (x,s))-f^*(\phi (x,s))|^2|\partial _s\phi (x,s)|dxds. \end{aligned}$$

Thanks to (2.29), we have

2.34 $$\begin{aligned} f(x,\phi (x,s))&-f^*(\phi (x,s))\nonumber \\&=s- f^{*}(\phi _1(s)) + \left( f^*(\phi _1(s)) -f^*(\phi _1(s) + h(x,s))\right) \nonumber \\&= f^*(\phi _1(s)) -f^*(\phi _1(s) + h(x,s))\nonumber \\&= \partial _2 f^*(\phi _1(s))h(x,s) + O \left( |\partial _{22}f^*||h(x_1,s)|^2\right) \nonumber \\&=\partial _2\rho _s h(x,s) + O\left( |\partial _2(\rho _s-f^*)||h(x_1,s)|\right) +O \left( |\partial _{22}f^*||h(x,s)|^2\right) \nonumber \\&= \partial _2\rho _sh(x,s) + O(\delta |h(x,s)|), \end{aligned}$$

where the last equality follows from (2.32). Note that (2.30) also implies $|\partial _s\phi -\partial _s\phi _0|\le _C\delta$, thus (2.31) gives

2.35 $$\begin{aligned} 0 < \frac{1}{C}\le -\partial _s\phi \le C. \end{aligned}$$

Plugging this and (2.34) into (2.33), we obtain, for sufficiently small $\delta >0$,

2.36 $$\begin{aligned} C^{-1}\Vert h\Vert _{L^2(\mathbb {T}\times [0,1])}\le \Vert f-f^*\Vert _{L^2(\Omega )}\le C\Vert h\Vert _{L^2(\mathbb {T}\times [0,1])}. \end{aligned}$$

On the other hand, $E_P(f)$ can be written as

$$\begin{aligned} E_P(f)&= \int _{\mathbb {T}}\int _{0}^1 f(x_1,x_2)x_2 dx_2 \\&= -\int _{\mathbb {T}}\int _I f(x_1,\phi (x_1,s))\phi (x_1,s)\partial _{s}\phi (x_1,s)dsdx_1\\&= -\int _{\mathbb {T}}\int _I s \frac{1}{2}\partial _s\left( \phi (x_1,s) \right) ^2dsdx_1. \end{aligned}$$

Since $f=f^*=\rho _s$ on $\partial \Omega$ and $\rho _s$ is strictly decreasing, we have

$$ \phi (x_1,\rho _s(0))=\phi _1(\rho _s(0)) =0,\quad \phi (x_1,\rho _s(1)) = \phi _1(\rho _s(1)) = 1. $$

Using this, we can continue the computation above as

$$\begin{aligned} E_P(f)&= -\frac{1}{2}\int _{\mathbb {T}} \phi (x_1,\rho _s(0))^2 - \phi (x_1,\rho _s(1))^2dx_1 + \frac{1}{2}\int _{\mathbb {T}}\int _I (\phi _1(s)+h(x_1,s))^2dsdx_1\\&=\pi + \frac{1}{2}\int _{\mathbb {T}}\int _0^1 \phi _1(s)^2 +h(x_1,s)^2dsdx_1, \end{aligned}$$

where the last equality follows from that *h* has zero average in *x*. Similarly, we have

$$\begin{aligned} E_P(f^*)&= \int _{\mathbb {T}}\int _0^1 f^*(x_2)dx = - \int _{\mathbb {T}}\int _I f^*(\phi _1(s))\phi _1(s)\partial _s\phi _1(s)dsdx_1 \\&= \pi + \frac{1}{2}\int _{\mathbb {T}}\int _I \phi _1(s)^2ds. \end{aligned}$$

Consequently, we arrive at $E(f) -E(f^*) = \frac{1}{2}\int _{\mathbb {T}}\int _I |h(x_1,s)|^2 dsdx_1.$ Combining this with (2.36), the estimates in (2.27) is verified.

Now, let us prove (2.28). Differentiating (2.29) in $x_1$, we see that

$$ 0 = \partial _1 (f(x_1,\phi (x_1,s))) = \partial _1f(x_1,\phi (x,s))+\partial _2f(x_1,\phi (x_1,s))\partial _1h(x_1,s). $$

Similarly, diffierentiating (2.29) in *s* yields $1= \partial _2 f(x_1,\phi (x_1,s))\partial _s\phi (s)$, thus

$$ \partial _1 f(x_1,\phi (x_1,s)) =-\frac{\partial _1h(x_1,s)}{\partial _s\phi (x_1,s)}. $$

Then, using the change of variables $x_2\rightarrow \phi (x_1,s)$ and also using (2.35), we obtain

$$ \Vert \partial _1 f\Vert _{L^2(\Omega )}\ge C\Vert \partial _1h\Vert _{L^2(\mathbb {T}\times I)}\ge C\Vert h\Vert _{L^2(\mathbb {T}\times I)}, $$

where the last inequality follows from the zero-average in *x* of *h* in (2.29) and the Poincaré inequality. Therefore, combining this with (2.36), we conclude that (2.28) holds.

## Stability in the IPM equation

In this section, we aim to prove the asymptotic stability for the incompressible porous media equation (1.2). Throughout the section, we will fix

3.1 $$\begin{aligned} \rho _s(x_2):=1-x_2. \end{aligned}$$

### Preliminaries for the IPM

Let us review important previous results concerning the local existence of the IPM equation (1.2) in the domain $\Omega =\mathbb {T}\times (0,1)$. For further details, we refer readers to the paper by Castro–Córdoba–Lear [2].

We recall the following spaces from [2, Section 1]: For $k\in \mathbb {N}$,

3.2 $$\begin{aligned} X^k(\Omega )&:=\left\{ f\in H^k(\Omega ) :\partial _{2}^nf|_{\partial \Omega }=0,\text { for }n=0,2,4,.\ldots k^\star \right\} ,\nonumber \\&\text { where }k^\star :={\left\{ \begin{array}{ll} k-2, & \text { if }k\text { is even},\\ k-1 &  \text { if }k\text { is odd}. \end{array}\right. } \end{aligned}$$

That is, $X^k(\Omega )$ is the closure of $\left\{ f\in C^\infty ({\Omega }):\partial _{2}^nf|_{\partial \Omega }=0,\text { for }n=0,2,4,.\ldots k^\star \right\}$ in the norm $H^k$. It is worth noting that the usual trace theorem, $H^k(\Omega )\hookrightarrow H^{k-1}(\partial \Omega )$, ensures that the vanishing normal derivatives in the above definition is well-defined. For convenience, we denote

$$ X^\infty (\Omega ):=\cap _{k\in \mathbb {N}}X^k(\Omega ). $$

By definition, it is clear from (2.1) that

3.3 $$\begin{aligned} C^\infty _0(\Omega )\subset X^\infty (\Omega ). \end{aligned}$$

If $f\in X^\infty (\Omega )$, it holds that

3.4 $$\begin{aligned} \partial _1^{n_1}\partial _2^{2n_2}f\in X^\infty (\Omega ),\text { for any }n_1,n_2\in \mathbb {N}\cup \left\{ 0\right\} , \end{aligned}$$

where $\partial _n$ denotes the normal derivative of *f* on $\partial _\Omega$. Especially, we can use the integration by parts in the vertical variable without a boundary integral:

3.5 $$\begin{aligned} \int _{\Omega } \partial _2^{k+1}f(x)\partial _2^{k+1}g(x)dx&= - \int _{\Omega }\partial _2^{k}f(x)\partial _2^{k+2}g(x)dx, \nonumber \\&\text { for any } k\in \mathbb {N}\cup \left\{ 0\right\} \text { and }f,g\in X^\infty (\Omega ). \end{aligned}$$

In a usual domain without boundary, for instance $\mathbb {R}^2$, it is trivial that the Sobolev norms can be bounded by looking at only each single directional derivatives, that is,

$$ \Vert f\Vert _{\dot{H}^k(\mathbb {R}^2)}\le C\left( \Vert \partial _1^{k}f\Vert _{L^2(\mathbb {R}^2)}+\Vert \partial _2^{k}f\Vert _{L^2(\mathbb {R}^2)}\right) . $$

In a bounded domain, this property may depend on the boundary condition, since a mixed derivative might not be well controlled. While the next lemma seems intuitively obvious, we will give a proof for the sake of completeness, although the proof will be postponed to Appendix 5.

#### Lemma 3.1

Let $f\in X^\infty (\Omega )$. For any $n,k\in \mathbb {N}\cup \left\{ 0\right\}$, we have

3.6 $$\begin{aligned} \Vert \partial _{1}^n\partial _2^kf\Vert _{L^2(\Omega )}\le C_{n,k}\left( \Vert \partial _1^{n+k}f\Vert _{L^2(\Omega )} +\Vert \partial _2^{n+k}f\Vert _{L^2(\Omega )} \right) . \end{aligned}$$

Consequently, we have

3.7 $$\begin{aligned} \Vert f\Vert _{{H}^k}\le C_{k}\left( \Vert \partial _1^kf\Vert _{L^2} + \Vert \partial _2^k f\Vert _{L^2}\right) \text { for all }k\ge 0. \end{aligned}$$

Let us consider a solution $\rho (t)$ to the IPM equation. We denote

3.8 $$\begin{aligned} \theta (t):=\rho (t)-\rho _s. \end{aligned}$$

Substituting $\rho =\theta +{\rho }_s$ in (1.2), one can easily see that $\theta (t)$ solves

3.9 $$\begin{aligned} {\left\{ \begin{array}{ll} \theta _t + u\cdot \nabla \theta = u_2,\\ u=\nabla ^\perp \Psi , \end{array}\right. } \text { with } {\left\{ \begin{array}{ll} -\Delta \Psi = \partial _1\theta , &  \text { in }\Omega ,\\ \Psi = 0, &  \text { on }\partial \Omega . \end{array}\right. } \end{aligned}$$

The next lemma tells us that if the solution $\theta (t)\in X^k$, then the stream function $\Psi (t)$ behaves in a similar manner.

#### Lemma 3.2

[2, Lemma 3.1] Let $f\in X^k(\Omega )$ and let $\Psi$ be a solution to

$$ {\left\{ \begin{array}{ll} \Delta \Psi = - \partial _1f &  \text { in }\Omega ,\\ \Psi = 0 &  \text { on }\partial \Omega . \end{array}\right. } $$

Then $\Psi \in X^{k+1}(\Omega )$ and it satisfies $\Vert \Psi \Vert _{H^{k+1}}\le C_k \Vert f\Vert _{H^k}$.

The local well-posedness to the equation (3.9) was established in [2]:

#### [Style2 Style2]Theorem 3.3

[2, Theorem 4.1] Let $k\in \mathbb {N}$ with $k\ge 3$. For any $\theta _0\in X^k(\Omega )$, there exists a time $T=T(\Vert \theta _0\Vert _{H^3})>0$ and a unique solution $\theta \in C(0,T; X^k(\Omega ))$ for the equation (3.9).

Thanks to the local well-posedness theorem, we will assume that the initial data is smooth, that is, $\theta _0\in X^\infty$. In view of the statement of Theorem 1.1, the general case where $\theta _0\in H^k_0(\Omega )$ will be managed by usual compactness argument in the end of the section.

### Energy estimates

In this section, we aim to derive an a priori energy estimate. The main result will be given in Proposition 3.6.

Let us recall some well-known estimates concerning the Sobolev spaces. In the next lemma, we use the following notations: For $\alpha \in (\mathbb {N}\cup \left\{ 0\right\} )^2$,

$$ \alpha =(\alpha _1,\alpha _2),\quad |\alpha |:=\alpha _1+\alpha _2,\quad \partial ^{\alpha }:=\partial _1^{\alpha _1}\partial _2^{\alpha _2}. $$

#### Lemma 3.4

[2, Lemma 4.2] Let $f,g\in C^\infty (\Omega )$. Then, for $\alpha ,\beta \in (\mathbb {N}\cup \left\{ 0\right\} )^2$, we have

$$\begin{aligned} \Vert \partial ^\alpha f\partial ^\beta g\Vert _{L^2}&\le _{\alpha ,\beta } \Vert f\Vert _{H^{|\alpha |+|\beta |}}\Vert g\Vert _{L^\infty } +\Vert g\Vert _{H^{|\alpha |+|\beta |}}\Vert f\Vert _{L^\infty },\\ \Vert \partial ^\alpha (fg)-f\partial ^\alpha g\Vert _{L^2}&\le _{\alpha ,\beta } \Vert f\Vert _{H^{|\alpha |}}\Vert g\Vert _{L^\infty }+\Vert f\Vert _{W^{1,\infty }}\Vert g\Vert _{H^{|\alpha |-1}}. \end{aligned}$$

#### Lemma 3.5

For $f\in H^1(\Omega )$ and $\overline{f}(x_2):=\frac{1}{2\pi }\int _{\mathbb {T}} f(x_1,x_2)dx_1$, it holds that

$$ \Vert f-\overline{f}\Vert _{L^\infty }\le _C \Vert \partial _1f\Vert _{H^1}. $$

#### Proof

We notice the following pointwise estimate:

3.10 $$\begin{aligned} |(f-\overline{f})(x)|^2 = \left( \frac{1}{2\pi }\int _{\mathbb {T}}f(x_1,x_2)-f(z,x_2)dz\right) ^2\le C\int _{\mathbb {T}}(f(x_1,x_2)-f(z,x_2))^2dz. \end{aligned}$$

The integrand in the right-hand side can be written as $f(x_1,x_2)-f(z,x_2) = \int _{x_1}^{z}\partial _{1}f(a,x_2)da.$ For each fixed $x_1$, we apply the Sobolev embedding $H^1([0,1])\hookrightarrow L^\infty ([0,1])$ to the map $x_2\mapsto \int _{x_1}^{z}\partial _{1}f(a,x_2)da$, yielding that

$$ \sup _{x_2\in [0,1]}\left| \int _{x_1}^{z}\partial _1f(a,x_2)da\right| ^2\le C\int _{0}^{1}\int _{x_1}^{z}|\partial _{12}f(a,y)|^2 + |\partial _{1}f(a,y)|^2dady\le \Vert \partial _1f\Vert _{H^1}^2. $$

Therefore, taking the supremum over $x\in \Omega$ in (3.10), the desired result follows.

#### Proposition 3.6

Let $\theta _0\in C^\infty _0(\Omega )$ and $\theta (t)\in C(0,T; X^\infty (\Omega ))$ be the unique smooth solution to (3.9) for some $T>0$. For $k\ge 3$, it holds that

$$\begin{aligned} \frac{d}{dt}\left( \Vert \partial _1^k \theta \Vert _{L^2}^2 + \Vert \partial _2^k \theta \Vert _{L^2}^2 \right)&\le -C_k(1-C_k\Vert \theta \Vert _{H^k})\Vert \nabla \Psi \Vert _{{H}^k}^2 + C_k\Vert u_2\Vert _{W^{1,\infty }}\Vert \theta \Vert _{H^k}^2. \end{aligned}$$

#### Proof

In the proof, the implicit constant *C* may depend on *k*, but its dependence will be omitted for simplicity. In what follows $\partial _i$ will denote either $\partial _1$ or $\partial _2$. Using (3.9), we compute

3.11 $$\begin{aligned} \frac{1}{2}\frac{d}{dt}\Vert \partial _i^k \theta \Vert _{L^2}^2 = -\int \partial _i^k(u\cdot \nabla \theta ) \partial _i^k\theta dx +\int \partial _i^ku_2\partial _i^k\theta dx \end{aligned}$$

We simplify the linear term first. Recalling from (3.9) that $u_2=\partial _1\Psi$ and $\partial _1\theta =-\Delta \Psi$, we have

$$\begin{aligned} \int \partial _i^ku_2\partial _i^k\theta dx&=\int \partial _1\partial _i^k\Psi \partial _i^k\theta dx = -\int \partial _i^k\Psi \partial _i^k\partial _1\theta dx = \int \partial _i^k\Psi \partial _i^k\Delta \Psi dx\nonumber \\&= \int _{\partial \Omega }\partial _i^k\Psi \nabla (\partial _i^k \Psi )\cdot \vec {n}(x)d\sigma (x) - \int _{\Omega }|\nabla \partial _i^k\Psi |^2dx= - \int _{\Omega }|\nabla \partial _i^k\Psi |^2dx, \end{aligned}$$

where the last equality follows from (3.5), which ensures that the integral over $\partial \Omega$ vanishes. Since $\theta \in X^\infty (\Omega )$, it follows from Lemma 3.2 and Lemma 3.1 that $\sum _{i=1,2}\int _{\Omega }|\nabla \partial _i^k\Psi |^2dx\ge _C \Vert \nabla \Psi \Vert _{\dot{H}^k}^2$. Since $\Psi =0$ on $\partial \Omega$, the Poincaré inequality gives us $\Vert \nabla \Psi \Vert _{\dot{H}^k}^2\ge _C \Vert \nabla \Psi \Vert _{H^k}$, consequently,

3.12 $$\begin{aligned} \sum _{i=1,2}\int \partial _i^ku_2\partial _i^k\theta dx \le _C -\Vert \nabla \Psi \Vert _{{H}^k}^2. \end{aligned}$$

Now, we move on to the nonlinear term. We write

3.13 $$\begin{aligned} \int \partial _i^k(u\cdot \nabla \theta ) \partial _i^k\theta dx&=\int \left( \partial _i^k(u\cdot \nabla \theta )-u\nabla \partial _i^k\theta \right) \partial _i^k\theta dx + \int u\cdot \nabla \partial _i^k \theta \partial _i^k\theta dx \nonumber \\&= \int \left( \partial _i^k(u\cdot \nabla \theta )-u\nabla \partial _i^k\theta \right) \partial _i^k\theta dx + \int u\cdot \nabla \left( \frac{1}{2}(\partial _i^k\theta )^2\right) dx\nonumber \\&= \int \left( \partial _i^k(u\cdot \nabla \theta )-u\nabla \partial _i^k\theta \right) \partial _i^k\theta dx, \end{aligned}$$

where the last equality follows from the integration by parts and $u\cdot \vec {n}=0$ on the boundary. We claim that

3.14 $$\begin{aligned} \left| \int \left( \partial _i^k(u\cdot \nabla \theta )-u\nabla \partial _i^k\theta \right) \partial _i^k\theta dx\right| \le C\left( \Vert u_2\Vert _{W^{1,\infty }}\Vert \theta \Vert _{H^k}^2 +\Vert \nabla \Psi \Vert _{{H}^k}^2\Vert \theta \Vert _{H^k} \right) , \end{aligned}$$

either $i=1$ or $i=2$. Once we have the above claim, combining it with (3.12) yields the desired energy estimate. Thus, in the rest of the proof, we will aim to prove the claim (3.14). We consider two cases, $i=1$ and $i=2$, separately.

**Case **$i=1$. The Cauchy-Schwarz inequality gives us

3.15 $$\begin{aligned} \left| \int \left( \partial _1^k(u\cdot \nabla \theta )-u\nabla \partial _1^k\theta \right) \partial _1^k\theta dx\right| \le _C \Vert \partial _1^k(u\cdot \nabla \theta )-u\nabla \partial _1^k\theta \Vert _{L^2}\Vert \partial _1^k\theta \Vert _{L^2}, \end{aligned}$$

while Lemma 3.4 tells us that

$$ \Vert \partial _1^k(u\cdot \nabla \theta )-u\nabla \partial _1^k\theta \Vert _{L^2}\le C\left( \Vert u\Vert _{W^{1,\infty }}\Vert \theta \Vert _{H^k}+\Vert u\Vert _{H^k}\Vert \nabla \theta \Vert _{L^\infty }\right) . $$

Since $k\ge 3$, the Sobolev inequality gives us

3.16 $$\begin{aligned} \Vert u\Vert _{W^{1,\infty }}=\Vert \nabla \Psi \Vert _{W^{1,\infty }}\le C\Vert \nabla \Psi \Vert _{H^k},\text { and }\Vert \nabla \theta \Vert _{L^\infty }\le C\Vert \theta \Vert _{H^k}. \end{aligned}$$

Hence we have $\Vert \partial _1^k(u\cdot \nabla \theta )-u\nabla \partial _1^k\theta \Vert _{L^2}\le C\Vert \nabla \Psi \Vert _{H^k}\Vert \theta \Vert _{H^k}$. Plugging this into (3.15), we obtain

$$ \left| \int \left( \partial _1^k(u\cdot \nabla \theta )-u\nabla \partial _1^k\theta \right) \partial _1^k\theta dx\right| \le C \Vert \nabla \Psi \Vert _{H^k}\Vert \partial _1^k\theta \Vert _{L^2}\Vert \theta \Vert _{H^k}. $$

Furthermore, from the Poisson equation in (3.9), we find that

$$ \Vert \partial _1^k\theta \Vert _{L^2} = \Vert \partial _1^{k-1}\Delta \Psi \Vert _{L^2}\le C\Vert \nabla \Psi \Vert _{H^{k}}, $$

therefore, we conclude

3.17 $$\begin{aligned} \left| \int \left( \partial _1^k(u\cdot \nabla \theta )-u\nabla \partial _1^k\theta \right) \partial _1^k\theta dx\right| \le C \Vert \nabla \Psi \Vert _{H^k}^2\Vert \theta \Vert _{H^k}, \end{aligned}$$

which verifies (3.14).

**Case **$i=2$. Splitting $u\cdot \nabla =u_1\partial _1+u_2\partial _2$, we have

3.18 $$\begin{aligned} \int \left( \partial _2^k(u\cdot \nabla \theta )-u\nabla \partial _2^k\theta \right) \partial _2^k\theta dx&= \int \left( \partial _2^k(u_1\partial _1 \theta )-u_1 \partial _2^k\partial _1\theta \right) \partial _2^k\theta dx \nonumber \\&+ \int \left( \partial _2^k(u_2 \partial _2\theta )-u_2 \partial _2^k\partial _2\theta \right) \partial _2^k\theta dx\nonumber \\&=:I_1+I_2. \end{aligned}$$

The first integral $I_1$ can be estimated as before; applying the Cauchy-Schwarz inequality, we get

3.19 $$\begin{aligned} |I_1|\le C\Vert \partial _2^k(u_1\partial _1\theta )-u_1\partial _2^k\partial _1\theta \Vert _{L^2}\Vert \partial _2^k\theta \Vert _{L^2}, \end{aligned}$$

while Lemma 3.4 gives us

$$\begin{aligned} \Vert \partial _2^k(u_1\partial _1\theta )-u_1\partial _2^k\partial _1\theta \Vert _{L^2}&\le C\left( \Vert u_1\Vert _{W^{1,\infty }}\Vert \partial _1\theta \Vert _{H^{k-1}} +\Vert u_1\Vert _{H^k}\Vert \partial _1\theta \Vert _{L^\infty }\right) \\&\le C\Vert \nabla \Psi \Vert _{H^k}\left( \Vert \partial _1\theta \Vert _{H^{k-1}}+\Vert \partial _1\theta \Vert _{L^\infty }\right) , \end{aligned}$$

where we used the estimate for *u* in (3.16) to get the second inequality. Again, using the Sobolev inequality and the Poisson equation in (3.9), we estimate

$$ \Vert \partial _1\theta \Vert _{L^\infty }\le _C \Vert \partial _1\theta \Vert _{H^{k-1}}=\Vert \Delta \Psi \Vert _{H^{k-1}} \le \Vert \nabla \Psi \Vert _{H^{k}}, $$

which gives us $\Vert \partial _2^k(u_1\partial _1\theta )-u_1\partial _2^k\partial _1\theta \Vert _{L^2}\le \Vert \nabla \Psi \Vert _{H^{k}}^2$. Plugging this into (3.19), we conclude

3.20 $$\begin{aligned} |I_1|\le C\Vert \nabla \Psi \Vert _{H^k}^2\Vert \partial _2^k\theta \Vert _{L^2}\le C\Vert \nabla \Psi \Vert _{H^k}^2\Vert \theta \Vert _{H^k}. \end{aligned}$$

Now, let us estimate $I_2$ in (3.18). We denote

3.21 $$\begin{aligned} \overline{\theta }(x_2):=\frac{1}{2\pi }\int _{\mathbb {T}}\theta (x_1,x_2)dx_1. \end{aligned}$$

We split $I_2$ as

3.22 $$\begin{aligned} I_2&= \int \left( \partial _2^k(u_2 \partial _2(\theta -\overline{\theta }))-u_2 \partial _2^k\partial _2(\theta -\overline{\theta })\right) \partial _2^k\theta dx \nonumber \\  &+ \int \left( \partial _2^k(u_2 \partial _2\overline{\theta })-u_2 \partial _2^k\partial _2\overline{\theta }\right) \partial _2^k\theta dx=: I_{21} + I_{22} \end{aligned}$$

We estimate $I_{21}$ first. In a similar manner as above, the Cauchy-Schwarz inequality and the Sobolev inequalities yield

3.23 $$\begin{aligned} |I_{21}|&\le _C\left( \Vert u_2\Vert _{W^{1,\infty }}\Vert \partial _2(\theta -\overline{\theta })\Vert _{H^{k-1}} +\Vert u_2\Vert _{H^k}\Vert \partial _2(\theta -\overline{\theta })\Vert _{L^\infty }\right) \Vert \theta \Vert _{H^k}\nonumber \\&\le _C\left( \Vert u_2\Vert _{W^{1,\infty }}\Vert (\theta -\overline{\theta })\Vert _{H^{k}} +\Vert u_2\Vert _{H^k}\Vert \partial _2(\theta -\overline{\theta })\Vert _{L^\infty }\right) \Vert \theta \Vert _{H^k}\nonumber \\&\le _C\Vert u_2\Vert _{W^{1,\infty }}\Vert \theta \Vert _{H^k}^2 + \Vert u_2\Vert _{H^k}\Vert \partial _2(\theta -\overline{\theta })\Vert _{L^\infty }\Vert \theta \Vert _{H^k}. \end{aligned}$$

Moreover, Lemma 3.5 implies

$$ \Vert \partial _2(\theta -\overline{\theta })\Vert _{L^\infty }\le _C \Vert \partial _{12}\theta \Vert _{H^1}\le _C \Vert \partial _2\Delta \Psi \Vert _{L^2}\le _C \Vert \nabla \Psi \Vert _{H^k}, $$

where the last inequality follows from $k\ge 3$. Plugging this and $\Vert u\Vert _{H^k}\le \Vert \nabla \Psi \Vert _{H^k}$ into (3.23), we obtain

3.24 $$\begin{aligned} |I_{21}|\le C\left( \Vert u_2\Vert _{W^{1,\infty }}\Vert \theta \Vert _{H^k}^2 + \Vert \nabla \Psi \Vert _{H^k}^2\Vert \theta \Vert _{H^k}\right) . \end{aligned}$$

Next, we estimate $I_{22}$ in (3.22). By expanding $I_{22}$ using the product rule, we have

3.25 $$\begin{aligned} I_{22}=\sum _{j=1}^{k}C_{k,j}\int \partial _{2}^ju_2\partial _2^{k-j+1}\overline{\theta }\partial _2^k\theta dx. \end{aligned}$$

When $j=1$, we have

3.26 $$\begin{aligned} \int \partial _{2}u_2\partial _2^{k}\overline{\theta }\partial _2^k\theta dx\le _C \Vert \partial _2u_2\Vert _{L^\infty }\Vert \overline{\theta }\Vert _{H^k}\Vert \theta \Vert _{H^k}\le _C \Vert \partial _2u_2\Vert _{L^\infty }\Vert \theta \Vert _{H^k}^2. \end{aligned}$$

For $j\ge 2$, noting that $u_2=\partial _1\Psi$, we can apply the integration by parts in each integral as

$$\begin{aligned} \int \partial _{2}^ju_2\partial _2^{k-j+1}\overline{\theta }\partial _2^k\theta dx&= -\int \partial _2^j\Psi \partial _2^{k-j+1}\overline{\theta } dx\partial _1\partial _2^k\theta dx\\&=-\int _{\partial \Omega }\partial _2^j\Psi \partial _2^{k-j+1}\overline{\theta }\partial _1\partial _2^{k-1}\theta d\sigma (x) \\&\quad +\int _{\Omega }\partial _2\left( \partial _2^j\Psi \partial _2^{k-j+1}\overline{\theta } \right) \partial _1\partial _2^{k-1}\theta dx\\&= \int _{\Omega }\partial _2\left( \partial _2^j\Psi \partial _2^{k-j+1}\overline{\theta } \right) \partial _1\partial _2^{k-1}\theta dx. \end{aligned}$$

To see the last inequality, note that by Lemma 3.2, we have that $\Psi ,\overline{\theta },\partial _1\theta$ are all in $X^\infty$. Since at least one of $j, k-j+1, k-1$ must be even, the definition of the space $X^k(\Omega )$ in (3.2) tells us that the boundary integral must vanish.

To continue, we apply the Cauchy-Schwarz inequality to get

3.27 $$\begin{aligned} \left| \int \partial _{2}^ju_2\partial _2^{k-j+1}\overline{\theta }\partial _2^k\theta dx\right|&=\left| \int _{\Omega }\partial _2\left( \partial _2^j\Psi \partial _2^{k-j+1}\overline{\theta } \right) \partial _1\partial _2^{k-1}\theta dx\right| \nonumber \\&\le \left( \Vert \partial _{2}^{j+1}\Psi \partial _2^{k-j+1}\overline{\theta }\Vert _{L^2}+\Vert \partial _2^j\Psi \partial _2^{k-j+2}\overline{\theta }\Vert _{L^2}\right) \Vert \partial _1\partial _2^{k-1}\theta \Vert _{L^2}\nonumber \\&\le \left( \Vert \partial _{2}^{j+1}\Psi \partial _2^{k-j+1}\overline{\theta }\Vert _{L^2}+\Vert \partial _2^j\Psi \partial _2^{k-j+2}\overline{\theta }\Vert _{L^2}\right) \Vert \nabla \Psi \Vert _{H^k}, \text { for }j\ge 2, \end{aligned}$$

where we used $\Vert \partial _1\partial _2^{k-1}\theta \Vert _{L^2} =\Vert \partial _{2}^{k-1}\Delta \Psi \Vert _{L^2}\le \Vert \nabla \Psi \Vert _{H^k}$ to get the last inequality.

Let us estimate $\Vert \partial _{2}^{j+1}\Psi \partial _2^{k-j+1}\overline{\theta }\Vert _{L^2}$ in (3.27). Since $2\le j\le k$, we have

3.28 $$\begin{aligned} \Vert \partial _{2}^{j+1}\Psi \partial _2^{k-j+1}\overline{\theta }\Vert _{L^2}\le \Vert \nabla \Psi \Vert _{H^{k}}\Vert \partial _2^{k-j+1}\overline{\theta }\Vert _{L^\infty }\le \Vert \nabla \Psi \Vert _{H^{k}(\Omega )}\Vert \overline{\theta }\Vert _{H^{k-j+2}}\le \Vert \nabla \Psi \Vert _{H^{k}}\Vert \theta \Vert _{H^{k}}, \end{aligned}$$

where the second last inequality follows from the Sobolev inequality, noticing that $\overline{\theta }$ depends only on the variable $x_2$.

Let us estimate $\Vert \partial _2^j\Psi \partial _2^{k-j+2}\overline{\theta }\Vert _{L^2}$ in (3.27). When $j=k$, we have

$$ \Vert \partial _2^j\Psi \partial _2^{k-j+2}\overline{\theta }\Vert _{L^2}\le _C \Vert \Psi \Vert _{H^k} \Vert \partial _{22}\overline{\theta }\Vert _{L^\infty }\le _C \Vert \nabla \Psi \Vert _{H^k}\Vert \theta \Vert _{H^k}. $$

When $j\le k-1$, we have

$$ \Vert \partial _2^j\Psi \partial _2^{k-j+2}\overline{\theta }\Vert _{L^2}\le \Vert \partial _2^j\Psi \Vert _{L^\infty }\Vert \partial _2^{k-j+2}\theta \Vert _{L^2}\le \Vert \nabla \Psi \Vert _{H^k}\Vert \theta \Vert _{H^k}. $$

where in the the last inequality, we used the Sobolev inequality and $j\ge 2$. Thus, we obtain

$$ \Vert \partial _2^j\Psi \partial _2^{k-j+2}\overline{\theta }\Vert _{L^2}\le \Vert \nabla \Psi \Vert _{H^k}\Vert \theta \Vert _{H^k},\text { for all }j\ge 2. $$

Plugging this and (3.28) into (3.27), we get

$$ \left| \int \partial _{2}^ju_2\partial _2^{k-j+1}\overline{\theta }\partial _2^k\theta dx\right| \le C\Vert \nabla \Psi \Vert _{H^k}^2\Vert \theta \Vert _{H^k} \text { for }j\ge 2. $$

Combining this with (3.26) and plugging them into (3.25), we see that

$$ |I_{22}|\le _C \Vert \partial _2u_2\Vert _{L^\infty }\Vert \theta \Vert _{H^k}^2 +\Vert \nabla \Psi \Vert _{H^k}^2\Vert \theta \Vert _{H^k}. $$

Plugging this and (3.24) into (3.22), we get

$$ |I_2|\le _C\Vert u_2\Vert _{W^{1,\infty }}\Vert \theta \Vert _{H^k}^2 +\Vert \nabla \Psi \Vert _{H^k}^2\Vert \theta \Vert _{H^k}. $$

Plugging this and (3.20) into (3.18), we conclude

$$ \left| \int \left( \partial _2^k(u\cdot \nabla \theta )-u\nabla \partial _2^k\theta \right) \partial _2^k\theta dx\right| \le _C \Vert u_2\Vert _{W^{1,\infty }}\Vert \theta \Vert _{H^k}^2 +\Vert \nabla \Psi \Vert _{H^k}^2\Vert \theta \Vert _{H^k}. $$

Combining this with (3.17), we obtain (3.14).

### Analysis of the energy structure

The main objective in this subsection is to derive sufficient convergence rate of $\rho (t)$ to the equilibrium $\rho _0^*$, while $\rho$ stays close to ${\rho }_s$. We emphasize that $\theta (t)=\rho (t)-\rho _s$ will stay small but not necessarily decay. Thus it is important to distinguish the roles of $\theta (t)$ and $\rho (t)-\rho _0^*$. We will consider the potential energy and the kinetic energy defined as

3.29 $$\begin{aligned} E(t):= \int _{\Omega }(\rho (t)-\rho _0^*)x_2dx,\quad K(t):=\Vert u(t)\Vert _{L^2}^2. \end{aligned}$$

Since we are interested in a solution that is close to $\rho _s$, we will assume, throughout this subsection, that

3.30 $$\begin{aligned} \Vert \theta (t)\Vert _{H^3}^2+ \int _0^T \Vert \nabla \Psi (t)\Vert _{H^k}^2 dt\le \delta \le \delta _0, \text { for } t\in [0,T]\hbox { for some }T>0\hbox { and }\delta _0\ll 1. \end{aligned}$$

#### Proposition 3.7

Let $k\ge 3$. There exists $\delta _0=\delta _0(k)>0$ such that if $\rho (t)$ satisfies (3.30), then

3.31 $$\begin{aligned} \frac{d}{dt}E(t)= -K(t),\quad \frac{d}{dt}K(t)\le -C_k \Vert u_2\Vert _{L^2}^2. \end{aligned}$$

#### Proof

Differentiating *E*(*t*), we see that

3.32 $$\begin{aligned} \frac{d}{dt}E(t) = \int _{\Omega }\rho _t x_2dx = -\int _{\Omega }u\cdot \nabla \rho x_2dx = \int _{\Omega }u_2\rho dx. \end{aligned}$$

Using (1.3), we can further simplify the last expression as

$$ \int _{\Omega }u_2\rho dx = \int _{\Omega }\partial _1\Psi \rho dx = -\int _{\Omega }\Psi \partial _1\rho dx = \int _{\Omega }\Psi \Delta \Psi dx = -\int _{\Omega }|\nabla \Psi |^2 dx, $$

therefore, we get

3.33 $$\begin{aligned} \frac{d}{dt}E(t) =-\int _{\Omega }|\nabla \Psi |^2 dx = -K(t). \end{aligned}$$

In order to estimate $\frac{d}{dt}K(t)$, we differentiate (3.32) and obtain

$$\begin{aligned} \frac{1}{2}\left( \frac{d}{dt}\right) ^2 E(t)&=\frac{1}{2} \frac{d}{dt}\int _{\Omega } u_2\rho dx = \frac{1}{2}\int _{\Omega } u_2 \rho _t dx \\  &\quad + \frac{1}{2}\int _{\Omega }\partial _tu_2 \rho dx. \end{aligned}$$

Using $u_2=\partial _1\Psi$ and $\partial _1\rho =\Delta \Psi$, we have

$$\begin{aligned} \int _{\Omega }\partial _tu_2 \rho dx&= -\int _{\Omega }\partial _t\Psi \partial _1\rho dx = \int _{\Omega }\partial _t\Psi \Delta \Psi dx= \int _{\Omega }\partial _t\Delta \Psi \Psi dx \\  &= -\int _{\Omega }{\partial _t}\partial _1\rho \Psi dx = \int _{\Omega }\partial _t\rho u_2 dx \end{aligned}$$

Therefore, we get

$$\begin{aligned} \frac{1}{2}\left( \frac{d}{dt}\right) ^2 E(t)&= \int _{\Omega }u_2\rho _t = -\int _{\Omega } u_2 u \cdot \nabla \rho dx = -\int _{\Omega }u_2^2\partial _2\rho dx - \int _{\Omega }u_2u_1\partial _1\rho dx\\&\ge -\int _{\Omega }u_2^2\partial _2\rho dx - \Vert u_2\Vert _{L^2}\Vert u_1\partial _1\rho \Vert _{L^2}.\end{aligned}$$

Using the assumption on $\Vert \theta \Vert _{H^3}$ in (3.30) and (3.1), we have

$$ -\int _{\Omega }u_2^2\partial _2\rho dx = -\int u_2^2 \partial _2\rho _s dx -\int u_2^2 \partial _2\theta dx \ge \Vert u_2\Vert _{L^2}^2- \sqrt{\delta _0}\Vert u_2\Vert _{L^2}^2\ge C\Vert u_2\Vert _{L^2}^2 . $$

Using (3.33), we see that the above inequality implies

3.34 $$\begin{aligned} C\frac{d}{dt}K(t) + \Vert u_2\Vert _{L^2}^2 \le C\Vert u_2\Vert _{L^2}\Vert u_1\partial _1\rho \Vert _{L^2}. \end{aligned}$$

Let us estimate $\Vert u_1\partial _1\rho \Vert _{L^2(\Omega )}$. Using (1.3), we rewrite

3.35 $$\begin{aligned} \Vert u_1\partial _1\rho \Vert _{L^2(\Omega )} = \Vert \partial _2\Psi \Delta \Psi \Vert _{L^2}\le \Vert \nabla \Psi \Vert _{L^4}\Vert \Delta \Psi \Vert _{L^4} \le \Vert \nabla \Psi \Vert _{H^3}\Vert \Psi \Vert _{L^2}, \end{aligned}$$

where the last inequality is due to the Gagliardo-Nirenberg interpolation theorem.  Moreover, we notice from (1.3) that $g(x_2):=\int _{\mathbb {T}}\Psi (x_1,x_2)dx_1$ satisfies

$$\begin{aligned} \partial _{22}g(x_2)&= \int _{\mathbb {T}}\partial _{22}\Psi (x_1,x_2)dx_1 = \int _{\mathbb {T}}\Delta \Psi (x_1,x_2) - \partial _{11}\Psi (x_1,x_2)dx_1\\  &=\int _{\mathbb {T}}\partial _1(-\rho +\partial _1\Psi )dx=0, \end{aligned}$$

with the boundary condition, $g(0)=g(1)=0$. Therefore, $g=0$ for all $x_2\in [0,1]$. In other words, the map $x_1\rightarrow \Psi (x_1,x_2)$ has zero average for each fixed $x_2$. Then, the Poincaré inequality tells us

$$ \Vert \Psi \Vert _{L^2}\le \Vert \partial _1\Psi \Vert _{L^2}=\Vert u_2\Vert _{L^2}. $$

Plugging this into (3.35), we get

$$ \Vert u_1\partial _1\rho \Vert _{L^2}\le \Vert \nabla \Psi \Vert _{H^3}\Vert u_2\Vert _{L^2}\le \Vert \theta \Vert _{H^3}\Vert u_2\Vert _{L^2}\le \sqrt{\delta _0} \Vert u_2\Vert _{L^2}, $$

where the second inequality follows from Lemma 3.2 and the last inequality follows from (3.30). Plugging this into (3.34), we obtain that for sufficiently small $\delta _0>0$, $\frac{d}{dt}K(t)\le - C\Vert u_2\Vert _{L^2}^2.$ Combining this with (3.33), we finish the proof of the proposition.

#### Corollary 3.8

Let $k\ge 3$. There exists $\delta _0=\delta _0(k)>0$ such that if (3.30) holds, then,

$$\begin{aligned} E(t)&\le \frac{C\delta }{t^k},\\ \frac{2}{t}\int _{t/2}^{t} K(s)ds&\le \frac{C\delta }{t^{k+1}}, \\ \frac{2}{t}\int _{t/2}^{t}\Vert u_2(s)\Vert _{L^2}^2 ds&\le \frac{C\delta }{t^{k+2}}, \end{aligned}$$

for all $$t\in [0,T]$$.

#### Proof

Thanks to Proposition 2.5, it holds that

3.36 $$\begin{aligned} C^{-1}\Vert \rho (t)-\rho _0^*\Vert _{L^2}^2\le E(t)\le C\Vert \rho (t)-\rho _0^*\Vert _{L^2}^2 . \end{aligned}$$

Now, using the Gagliardo-Nirenberg interpolation theorem, we observe that

$$ \Vert \partial _1\rho \Vert _{L^2(\Omega )} =\Vert \Delta \Psi \Vert _{L^2}\le C \Vert \nabla \Psi \Vert _{H^k}^{1/k}\Vert \nabla \Psi \Vert _{L^2}^{(k-1)/k}. $$

On the other hand, applying (2.28), we get $\Vert \partial _1\rho \Vert _{L^2}\ge C\Vert \rho -\rho _0^*\Vert _{L^2}$. Combining this with the above estimate, we find

$$ \Vert \nabla \Psi \Vert _{L^2}^2\ge C \left( \Vert \nabla \Psi \Vert _{H^k}^{-1/k}\Vert \rho -\rho _0^*\Vert _{L^2}\right) ^{2k/(k-1)}\ge C \Vert \nabla \Psi \Vert _{H^k}^{-2/(k-1)} E(t)^{k/(k-1)}. $$

where the last inequality follows from (3.36). Hence, the variation of the potential energy in (3.31) must satisfy

3.37 $$\begin{aligned} \frac{d}{dt}E(t)\le - C\left( \Vert \nabla \Psi \Vert _{H^k}^2\right) ^{-1/(k-1)} E(t)^{k/(k-1)}. \end{aligned}$$

Applying Lemma 2.1 with $\alpha =1/(k-1)>0$ and $n=k/(k-1)$ and $a(t)=\Vert \nabla \Psi \Vert _{H^k}^2$, we get

3.38 $$\begin{aligned} E(t)&\le \frac{C\delta }{t^k}. \end{aligned}$$

Applying Lemma 2.2 to (3.31) with $f(t)=E(t),\ g(t)=K(t),\ h(t)=C\Vert u_2(t)\Vert _{L^2}^2$, we get

$$ \frac{2}{t}\int _{t/2}^{t} K(s)ds\le \frac{C}{t^{k+1}},\text { and } \frac{2}{t}\int _{t/2}^{t}\Vert u_2(s)\Vert _{L^2}^2 ds \le \frac{C\delta }{t^{k+2}}. $$

Together with (3.38), we obtain the desired estimates.

### Proof of Theorem 1.1

Let $k\ge 3$ be fixed. Let $\delta _0$ be be fixed as in Proposition 3.7 . We claim that there exists $\epsilon _0(k)>0$ such that if $\theta _0:=\rho _0-{\rho }_s\in C^\infty _0(\Omega )$ and

$$\begin{aligned} \Vert \rho _0-{\rho }_s\Vert _{H^k}\le \epsilon \le \epsilon _0,\end{aligned}$$

then for all $t>0$, it holds that

3.39 $$\begin{aligned} \Vert \theta (t)\Vert _{H^k}^2 +\int _0^t \Vert \nabla \Psi (t)\Vert _{H^k}^2 dt< C\epsilon ^2,\text { for some }C=C(k)>0. \end{aligned}$$

Let us suppose for the moment that the claim is true. From Theorem 3.3, we know that the maximal existence time depends only on $\Vert \theta (t)\Vert _{H^3}$, thus, the solution $\rho$ exists globally in time. Also, if necessary, we can further assume that $\epsilon$ is small enough to ensure Corollary 3.8 is applicable, that is, the solution $\rho (t)$ satisfies

3.40 $$\begin{aligned} E(t)\le \frac{C\epsilon ^2}{t^k},\quad \frac{2}{t}\int _{t/2}^{t} K(s)ds\le \frac{C\epsilon ^2}{t^{k+1}}, \quad \frac{2}{t}\int _{t/2}^{t}\Vert u_2(s)\Vert _{L^2}^2 ds \le \frac{C\epsilon ^2}{t^{k+2}},\text { for all }t>0. \end{aligned}$$

In order to prove the theorem for $\rho _0\in H^k_0$ without assuming the smoothness, we can simply find an approximation $\rho _{0,n}$ such that $\rho _{0,n}-\overline{\rho }\in C^\infty _{0}(\Omega )$ such that $\rho _{0,n}\rightarrow \rho _0$ in $H^k$. Then (3.40) and (3.39) are satisfied by the global solutions $\rho _n(t)$, with initial data $\rho _{0,n}$. Since all estimates are uniform in *n*, the unique limit $\rho (t):=\lim _{n\rightarrow \infty }\rho _n(t)$ is the global in time solution satisfying the same estimates, that is,

$$ \Vert \rho (t)-\rho _s\Vert _{H^k}^2 \le \epsilon ^2\quad \int _{\Omega }(\rho (t)-\rho _0^*)(x)x_2dx \le C\epsilon ^2t^{-k},\text { for all }t>0. $$

Together with (2.27), we obtain all the desired estimates to establish Theorem 1.1.

In the rest of the proof, we aim to prove (3.39). Towards a contradiction, let $T^*>0$ be the first time that (3.39) breaks down, that is,

3.41 $$\begin{aligned} \Vert \theta (T^*)\Vert _{H^k(\Omega )}^2 +\int _0^{T^*} \Vert \nabla \Psi (T^*)\Vert _{H^k(\Omega )}^2 dt= M\epsilon ^2\ll 1, \end{aligned}$$

for some $M>0$, which will be chosen later. Towards a contradiction, let us denote

$$ f(t):= \Vert \partial _1^k \theta \Vert _{L^2}^2 + \Vert \partial _2^k \theta \Vert _{L^2}^2. $$

Since $\theta _0=\rho _0-\overline{\rho }\in C^\infty _0(\Omega )$, we have $\theta _0\in X^\infty (\Omega )$. Therefore it follows from Theorem 3.3 that $\theta (t)\in X^\infty (\Omega )$ for $t\in [0,T^*]$. Since $\theta (t)$ vanishes on the boundary, Lemma 3.1 tells us that

3.42 $$\begin{aligned} C^{-1}\Vert \theta (t)\Vert _{H^k}^2\le f(t)\le C\Vert \theta (t)\Vert _{H^k}^2\le CM\epsilon ^2,\quad f(0)\le C\epsilon ^2. \end{aligned}$$

Using the Sobolev embedding $W^{1,\infty }(\Omega )\hookrightarrow H^k(\Omega )$ for $k\ge 3$, we have

3.43 $$\begin{aligned} \Vert u_2\Vert _{W^{1,\infty }}\le \Vert u_2\Vert _{H^k} \le \Vert \nabla \Psi \Vert _{H^k}\Vert \le \Vert \theta \Vert _{H^k}\le f(t)^{1/2}, \end{aligned}$$

where the second last inequality follows from (). Using this and Proposition 3.6, we observe that *f*(*t*) satisfies

3.44 $$\begin{aligned} \frac{d}{dt}f(t)\le -C\Vert \nabla \Psi (t)\Vert _{H^k}^2 + Cf(t)^{3/2},\text { for }t\in [0,T^*]. \end{aligned}$$

From this we see that *f* satisfies $\frac{d}{dt}f(t)\le Cf^{3/2}$. Since $f(0)\le C\epsilon ^2$, one can easily find that

3.45 $$\begin{aligned} f(t)\le \frac{C}{(\epsilon ^{-1}-t)^2}, \text { for }t\in [0,T^*]. \end{aligned}$$

This implies that for sufficiently small $\epsilon >0$, it holds that

3.46 $$\begin{aligned} f(t)\le C\epsilon ^2,\text { for }t\in [0,2]. \end{aligned}$$

In other words, for (3.41) to happen, we must have

3.47 $$\begin{aligned} T^*> 2. \end{aligned}$$

Now we analyze the energy inequality in a more careful manner. Using the Gagliardo-Nirenberg interpolation theorem, we see that

$$ \Vert u_2\Vert _{W^{1,\infty }}\le \Vert u_2\Vert _{H^k}^{2/k}\Vert u_2\Vert _{L^2}^{1-2/k}\le \Vert \nabla \Psi \Vert _{H^k}^{2/k}\Vert u_2\Vert _{L^2}^{1-2/k}. $$

Hence, using Young’s inequality, we derive

$$\begin{aligned} \Vert u_2\Vert _{W^{1,\infty }}f(t)&\le \Vert \nabla \Psi \Vert _{H^k}^{2/k}\Vert u_2\Vert _{L^2}^{1-2/k}f(t) \\&\le \eta \Vert \nabla \Psi \Vert _{H^k}^2 + C_{\eta }f(t)^{k/(k-1)}\Vert u_2\Vert _{L^2}^{(k-2)/(k-1)}\\&\le \eta \Vert \nabla \Psi \Vert _{H^k}^2 + C_{\eta }(M\epsilon ^2)^{k/(k-1)}\Vert u_2\Vert _{L^2}^{(k-2)/(k-1)}, \text { for any }\eta >0. \end{aligned}$$

Substituting this into (3.44) with sufficiently small $\eta$ depending only on the implicit constant *C*, we arrive at

$$ f'(t)\le -C\Vert \nabla \Psi \Vert _{H^k}^2 +C(M\epsilon ^2)^{k/(k-1)}\Vert u_2\Vert _{L^2}^{(k-2)/(k-1)}, $$

Integrating the above in *t* over $[1,T^*]$, we obtain

3.48 $$\begin{aligned} f(T^*)-f(1) + C_k\int _{1}^{T^*}\Vert \nabla \Psi (t)\Vert _{H^k}^2dt \le C_k(M\epsilon ^2)^{k/(k-1)} \int _{1}^{T^*}\left( \Vert u_2(t)\Vert _{L^2}^2\right) ^{\frac{k-2}{2(k-1)}}dt. \end{aligned}$$

Thanks to $M\epsilon ^2\ll 1$ in (3.41), we apply Corollary 3.8 and obtain

$$ \frac{2}{t}\int _{t/2}^{t}\Vert u_2(s)\Vert _{L^2}^2 ds \le \frac{CM\epsilon ^2}{t^{k+2}},\text { for all }t\in [0,T^*]. $$

Since $k\ge 3$, we have $n:=k+2>1$ and $\alpha :=\frac{k-2}{2(k-1)}\in (1/n,1)$. Thus applying Lemma 2.3 with $f=\Vert u_2\Vert _{L^2}^2$ and $E=CM\epsilon ^2$, we get

$$ \int _{1}^{T^*}\left( \Vert u_2(t)\Vert _{L^2}^2\right) ^{\frac{k-2}{2(k-1)}}dt\le C(M\epsilon ^2)^{\frac{k-2}{2(k-1)}}. $$

Plugging this and (3.46) into (3.48), we obtain

$$\begin{aligned} f(T^*)&+ C\int _{0}^{T^*}\Vert \nabla \Psi (t)\Vert _{H^k}^2dt\le C\left( \int _0^1\Vert \nabla \Psi \Vert _{H^k}^2dt +\epsilon ^2 +(M\epsilon ^2)^{\frac{3k-2}{2(k-1)}}\right) \\  &\le C \epsilon ^2 + C(M\epsilon ^2)^{\frac{3k-2}{2(k-1)}}, \end{aligned}$$

where we used (3.43) to justify the last inequality. Finally using $f(T^*)\ge C \Vert \theta \Vert _{H^k}^2$, which is due to the last inequality in (3.43), we notice that for (3.41) to hold, we must have

$$\begin{aligned} M\epsilon ^2&= \Vert \theta (T^*)\Vert _{H^k}^2 +\int _0^{T^*} \Vert \nabla \Psi (T^*)\Vert _{H^k(\Omega )}^2 dt\le _C f(T^*) + \int _{0}^{T^*}\Vert \nabla \Psi (t)\Vert _{H^k}^2dt\\  &\le C \epsilon ^2 + C(M\epsilon ^2)^{\frac{3k-2}{2(k-1)}}. \end{aligned}$$

Since $k\ge 3$, we have $\frac{3k-2}{2(k-1)}>1$. Therefore our assumption $M\epsilon ^2\ll 1$ in (3.41) and the above estimate give us

$$ M\epsilon ^2\le C\epsilon ^2. $$

This leads to a contradiction with (3.41), if *M* is chosen strictly larger than some implicit constant *C*, which depends only on *k*. This finishes the proof of Theorem 1.1.

## Stability in the Stokes transport system

In this section, our goal is to prove the asymptotic stability for the Stokes transport system (1.4). Throughout the section, we will assume that $\rho _s=\rho _s(x_2)$ is a function that is independent of $x_1$, and satisfies

4.1 $$\begin{aligned} \gamma :=\inf _{x\in \Omega }\left( -\partial _2\rho _s(x_2)\right) >0,\quad \Vert \rho _s\Vert _{H^4(\Omega )} <\infty . \end{aligned}$$

In the rest of the paper, we allow the implicit constant *C* to depend on $\gamma$ and $\Vert \rho _s\Vert _{H^4}$, but we omit its dependence in the notation for simplicity. While the proof will exhibit a structure quite similar to that of the previous section, we will furnish enough details for the sake of completeness of the paper.

### Preliminaries for the Stokes transport system

Let us briefly review relevant results concerning the Stokes transport system in the periodic channel For more details, we refer readers to the paper by Dalibard–Guillod–Leblond [5] and additional references therein.

Let us consider a solution $\rho (t)$ to the Stokes transport system. As in the previous section we denote

4.2 $$\begin{aligned} \theta (t):=\rho (t)-\rho _s. \end{aligned}$$

Substituting $\rho =\theta +{\rho }_s$ in (1.4), it immediately follows that

4.3 $$\begin{aligned} {\left\{ \begin{array}{ll} \theta _t + u\cdot \nabla \theta = -\partial _2\rho _su_2,\\ \nabla \cdot u =0,\\ \end{array}\right. } with \ {\left\{ \begin{array}{ll} \Delta ^2\Psi = \partial _1\theta , &  \text { in }\Omega ,\\ \Psi =\nabla \Psi =0, &  \text { on }\partial \Omega . \end{array}\right. } \end{aligned}$$

Thanks to the Bilaplacian operator in the equation for the stream function, the velocity in the Stokes transport system is much regular compared to that of the IPM equation. More quantitative estimate can be found in the next lemma.

#### Lemma 4.1

[5, Lemma B.1] Let $f\in H^k(\Omega )$ for $k\ge -2$ and $\Psi$ be a solution to

$$ {\left\{ \begin{array}{ll} \Delta ^2 \Psi = f &  \text { in }\Omega ,\\ \Psi =\nabla \Psi = 0 &  \text { on }\partial \Omega . \end{array}\right. } $$

Then $$\Psi \in H_{0}^2(\Omega )\cap H^{k+4}(\Omega )$$ and $$\Vert \Psi \Vert _{H^{k+4}}\le C_k \Vert f\Vert _{H^k}$$.

Thanks to more regular nature of the velocity, the global in time existence of the solution follows in a standard way. More precise statement can be formulated as below.

#### [Style2 Style2]Theorem 4.2

[5, Lemma 2.1, Theorem A.1] Let $\theta _0\in H^k(\Omega )$ with $k\ge 3$. Then there exists a unique global solution $\rho (t)$ to (1.4) such that $\rho \in C(\mathbb {R}^+; H^m(\Omega ))$. Furthermore, denoting $\theta :=\rho -{\rho }_s$ and $\overline{\theta }(x_2):=\frac{1}{2\pi }\int _{\mathbb {T}}\theta (x_1,x_2)dx_1$, it holds that if $\theta _0=\partial _n\theta =\partial _n^2\overline{\theta }=0$ on $\partial \Omega$, then

$$ \theta (t)=\partial _2\theta (t)=\partial _2^2\overline{\theta }(t) = 0,\text { on }\partial \Omega . $$

In view of Theorem 1.6, we will consider initial data $\theta _0\in H^2_0(\Omega )\cap H^4(\Omega )$. The usual trace theorem ensures that $\theta ,\partial _2\theta$ and $\partial _{22}\overline{\theta }$ are indeed well-defined on the boundary $\partial \Omega$ and vanish identically as stated in the above theorem.

### Energy estimates

In this subsection, our objective is to derive an a priori energy estimate. The main result is presented in Proposition 4.4. As one can notice from the proposition, we will focus on estimating the evolution of $\Vert \Delta ^2\theta \Vert _{L^2}$, rather than directly examining $\Vert \theta \Vert _{H^4}$. The reason is to mitigate potential complications in the finer analysis of the anisotropic nature of the nonlinear term in the equation. As expected, the norm $\Vert \Delta ^2\theta \Vert _{L^2}$ is equivalent to $\Vert \theta \Vert _{H^4}$ for solutions vanishing on the boundary. Although the immediate equivalence between these two norms may not be apparent through elementary integration by parts, Lemma 4.1 provides a rigorous justification

#### Lemma 4.3

If $f\in H^4(\Omega )$ satisfies $f=\partial _2f =0$ on $\partial \Omega$ and $\int _{\Omega }f(x)dx=0$, then

$$ \Vert f\Vert _{{H}^2(\Omega )} \le C\Vert \Delta f\Vert _{L^2(\Omega )},\quad \Vert f\Vert _{H^4(\Omega )}\le C\Vert \Delta ^2f\Vert _{L^2(\Omega )}. $$

#### Proof

The only problematic part is to ensure that the mixed derivatives can be estimated by the Laplacian/Bilaplacian operator. The first inequality is trivial since $\Vert \Delta f\Vert _{L^2}^2 = \Vert \partial _{11}f\Vert _{L^2}^2 +\Vert \partial _{22}f\Vert _{L^2}^2+2\Vert \partial _{12}f\Vert _{L^2}^2$, which is strong enough to control all the second derivatives of *f*. For the second inequality, we simply apply Lemma 4.1 with $k=0$, yielding the desired result.

#### Proposition 4.4

Let $\theta _0\in H^2_0(\Omega )\cap H^4(\Omega )$ and $\theta (t)$ be the unique smooth solution to (4.3). It holds that

$$\begin{aligned} \frac{d}{dt}\left( \Vert \Delta ^2 \theta \Vert _{L^2}^2\right)&\le _C -C(1- C\Vert \Delta ^2 \theta \Vert _{L^2})\Vert \partial _1\Delta \theta \Vert _{L^2}^2 + \left( \Vert u_2\Vert _{H^3} + \Vert u_2\Vert _{W^{2,\infty }}\right) \Vert \Delta ^2\theta \Vert _{L^2}^2\\&\ + \Vert u_2\Vert _{H^3}\Vert \Delta ^2\theta \Vert _{L^2}+\Vert \partial _1\theta \Vert _{L^2}^2. \end{aligned}$$

#### Proof

Using (4.3), we compute

4.4 $$\begin{aligned} \frac{1}{2}\frac{d}{dt}\Vert \Delta ^2 \theta \Vert _{L^2}^2 = -\int \Delta ^2 (u\cdot \nabla \theta )\Delta ^2 \theta dx +\int \Delta ^2 (-\partial _2\rho _s u_2)\Delta ^2 \theta dx \end{aligned}$$

We simplify the linear term first. Let us write

$$\begin{aligned} \int \Delta ^2 (-\partial _2\rho _s u_2)\Delta ^2 \theta dx&= \int \left( \Delta ^2(-\partial _2\rho _s u_2)-(-\partial _2\rho _s)\Delta ^2 u_2\right) \Delta ^2 \theta dx \\  &+ \int (-\partial _2\rho _s)\Delta ^2 u_2\Delta ^2 \theta dx=: A_1+A_2. \end{aligned}$$

For $A_1$, the usual tame estimate yields

4.5 $$\begin{aligned} |A_1|&\le _C \Vert \Delta ^2(-\partial _2\rho _s u_2)-(-\partial _2\rho _s)\Delta ^2 u_2\Vert _{L^2}\Vert \Delta ^2\theta \Vert _{L^2}\nonumber \\&\le _C \left( \Vert \partial _2\rho _s\Vert _{L^\infty }\Vert u_2\Vert _{H^3}+ \Vert \partial _2\rho _s\Vert _{H^4}\Vert u_2\Vert _{L^\infty }\right) \Vert \Delta ^2\theta \Vert _{L^2}\nonumber \\&\le _C\Vert u_2\Vert _{H^3}\Vert \Delta ^2\theta \Vert _{L^2}. \end{aligned}$$

For $A_2$, we can write

4.6 $$\begin{aligned} A_2&= \int -\partial _2\rho _s \partial _1\Delta ^2\Psi \Delta ^2\theta dx = \int \partial _2\rho _s\partial _1\theta \partial _1\Delta ^2\theta dx = \int \Delta (\partial _2\rho _s \partial _1\theta ) \partial _1\Delta \theta dx\nonumber \\&= \int \partial _2\rho _s |\partial _1\Delta \theta |^2 dx + \int (\partial _{222}\rho _s\partial _1\theta + 2\partial _{22}\rho _s\partial _{12}\theta )\partial _1\Delta \theta dx=: A_{21}+A_{22}, \end{aligned}$$

while $A_{22}$ can be estimated as

$$\begin{aligned} |A_{22}|&\le _C \Vert \partial _{222}\rho _s\Vert _{L^\infty }\Vert \partial _1\theta \Vert _{H^1}\Vert \partial _1\Delta \theta \Vert _{L^2}\le C\Vert \partial _1\theta \Vert _{H^1}\Vert \partial _1\Delta \theta \Vert _{L^2}\\&\le _C \Vert \partial _1\theta \Vert _{L^2}^{1/2}\Vert \partial _1\theta \Vert _{H^2}^{3/2}\le \Vert \partial _1\theta \Vert _{L^2}^{1/2}\Vert \partial _1\Delta \theta \Vert _{L^2}^{3/2} \le \frac{\inf (-\partial _2\rho _s)}{4}A_{21} + C\Vert \partial _1\theta \Vert _{L^2}^2 \end{aligned}$$

where the last inequality follows from Young’s inequality. Thus, using (4.1), we observe that (4.6) can be written as

$$ A_2 \le - \frac{3}{4}\inf (-\partial _2\rho _s)\Vert \partial _1\Delta \theta \Vert _{L^2}^2 + C\Vert \partial _1\theta \Vert _{L^2}^2\le -C\Vert \partial _1\Delta \theta \Vert _{L^2}^2+ C\Vert \partial _1\theta \Vert _{L^2}^2. $$

Combining this with (4.5), we get

4.7 $$\begin{aligned} \int \Delta ^2 (-\partial _2\rho _s u_2)\Delta ^2 \theta dx&\le -C\Vert \partial _1\Delta \theta \Vert _{L^2}^2+C\left( \Vert u_2\Vert _{H^3}\Vert \Delta ^2\theta \Vert _{L^2}+\Vert \partial _1\theta \Vert _{L^2}^2\right) . \end{aligned}$$

where the last equality follows from Lemma 4.2, which ensures that the integral over $\partial \Omega$ appearing in the integration by parts vanishes.

Now we estimate the integral coming from the nonlinear term in (4.4). We derive a necessary estimate in a separate lemma. Indeed, Lemma 4.5 gives us that

4.8 $$\begin{aligned} \left| \int \Delta ^2(u\cdot \nabla \theta )\Delta ^2\theta dx\right| \le _C \left( \Vert u_2\Vert _{H^3} + \Vert u_2\Vert _{W^{2,\infty }}\right) \Vert \Delta ^2\theta \Vert _{L^2}^2 +\Vert u\Vert _{H^5}\Vert \partial _1\Delta \theta \Vert _{L^2}\Vert \Delta ^2 \theta \Vert _{L^2}. \end{aligned}$$

Using Lemma 4.1,

$$ \Vert u\Vert _{H^5}\le _C \Vert \Psi \Vert _{H^5}\le _C \Vert \partial _1\theta \Vert _{H^1}\le _C \Vert \partial _1\Delta \theta \Vert _{L^2}, $$

where the last inequality follows from Lemma 4.3. Plugging this into (4.8), and combining it with (4.7), we conclude the proposition.

#### Lemma 4.5

Let *u* be a smooth divergence free vector field such that $u=0$ on $\partial \Omega$ and *f* be a smooth scalar-valued function such that $f=\partial _2 f=\partial _{2}^2 \overline{f}=0$ on $\partial \Omega$ where $\overline{f}:=\frac{1}{2\pi }\int _{\mathbb {T}}f(x_1,x_2)dx_1$. Then, we have

$$\begin{aligned}&\left| \int _{\Omega }\Delta ^2(u\cdot \nabla f)\Delta ^2f dx\right| \le _C \left( \Vert u_2\Vert _{H^3} + \Vert u_2\Vert _{W^{2,\infty }}\right) \Vert \Delta ^2f\Vert _{L^2}^2 +\Vert u\Vert _{H^5}\Vert \partial _1\Delta f\Vert _{L^2}\Vert \Delta ^2 f\Vert _{L^2} \end{aligned}$$

#### Proof

We split the integral $\int _{\Omega } \Delta ^2(u\cdot \nabla f)\Delta ^2f dx$ as

$$\begin{aligned} \int _{\Omega } \Delta ^2(u\cdot \nabla f)\Delta ^2f dx&= \int _{\Omega } \left( \Delta ^2(u\cdot \nabla f) - u\cdot \nabla \Delta ^2f\right) \Delta ^2\theta + \int _{\Omega }u\cdot \nabla \left( \frac{1}{2}(\Delta ^2f)^2\right) dx\\&=\int _{\Omega } \left( \Delta ^2(u\cdot \nabla f ) - u\cdot \nabla \Delta ^2\theta \right) \Delta ^2f dx, \end{aligned}$$

where the last integral vanishes due to the incompressibility of *u* and the boundary condition $u=0$ on $\Omega$. Next, we further decompose the last integral as

4.9 $$\begin{aligned} \int _{\Omega } \Delta ^2(u\cdot \nabla f)\Delta ^2f dx&= \int _{\Omega } \left( \Delta ^2(u\cdot \nabla f) - u\cdot \nabla \Delta ^2f\right) \Delta ^2fdx\nonumber \\&= \int _{\Omega }\left( \Delta ^2(u_1\partial _1f) - u_1\Delta ^2\partial _1f\right) \Delta ^2f dx \nonumber \\&\quad + \int _{\Omega }\left( \Delta ^2(u_2\partial _2f) - u_2\Delta ^2\partial _2f\right) \Delta ^2f dx \nonumber \\&=: I_1 + I_2. \end{aligned}$$

**Estimate for **$I_1$. Using the Cauchy-Schwarz inequality, we estimate

$$ |I_1|\le \Vert \Delta ^2(u_1\partial _1f) - u_1\Delta ^2\partial _1f\Vert _{L^2}\Vert \Delta ^2f\Vert _{L^2}. $$

Using Lemma 3.4, we have

$$\begin{aligned} \Vert \Delta ^2(u_1\partial _1f) - u_1\Delta ^2\partial _1f\Vert _{L^2}&\le \Vert u_1\Vert _{H^4}\Vert \partial _1f\Vert _{L^\infty } +\Vert u_1\Vert _{W^{1,\infty }}\Vert \partial _1f\Vert _{H^3}\\&\le \Vert u_1\Vert _{H^4}\Vert \partial _1f\Vert _{H^2} +\Vert u_1\Vert _{W^{1,\infty }}\Vert \partial _1f\Vert _{H^3}. \end{aligned}$$

where the second inequality is due to the Sobolev embedding $H^2(\Omega )\hookrightarrow L^\infty (\Omega )$. Then, using Lemma 4.3, we can replace $\Vert \partial _1f\Vert _{H^2}$ and $\Vert \partial _1f\Vert _{H^3}$ in the above estimate by $\Vert \partial _1\Delta f\Vert _{L^2}$ and $\Vert \Delta ^2 f\Vert _{L^2}$, respectively. Hence, we obtain

4.10 $$\begin{aligned} |I_1|\le \left( \Vert u_1\Vert _{H^4}\Vert \partial _1\Delta f\Vert _{L^2} +\Vert u_1\Vert _{W^{1,\infty }}\Vert \Delta ^2 f\Vert _{L^2}\right) \Vert \Delta ^2f\Vert _{L^2}. \end{aligned}$$

Before we start estimating $I_2$, we consider the structure of the bi-Laplacian acting on a product of two functions. Expanding the bi-Laplacian using the formula $\Delta (fg)=\Delta g h + 2\nabla g\cdot \nabla h + g\Delta h$, we have

4.11 $$\begin{aligned} \Delta ^2(gh)-f\Delta ^2h&= \Delta (\Delta gh + 2\nabla g\cdot \nabla h +g\Delta h) - g\Delta ^2 h\nonumber \\&= \Delta ^2 g h + 2\nabla \Delta g \cdot \nabla h + \Delta g\Delta h \nonumber \\&\quad \ + 2\left( \nabla \Delta g\cdot \nabla h+ \nabla \partial _1 g\cdot \nabla \partial _1h + \nabla \partial _2g\cdot \nabla \partial _2h + \nabla g\cdot \nabla \Delta h \right) \nonumber \\&\quad \ + \Delta g\Delta h + \nabla g\cdot \nabla \Delta h \nonumber \\&=\Delta ^2g h + 4\nabla \Delta g\cdot \nabla h + 2{\left( \nabla \partial _1 g\cdot \nabla \partial _1h + \nabla \partial _2g\cdot \nabla \partial _2h + \Delta g\cdot \Delta h \right) } \nonumber \\&\quad + \nabla g\cdot \nabla \Delta h . \end{aligned}$$

**Estimate for **$I_2$. Next, we move on to estimate $I_2$ in (4.9). Recalling our notation that $\overline{f}(x_2):=\frac{1}{2\pi }\int _{\mathbb {T}}f(x_1,x_2)dx_2,$ we split $I_2$ as

4.12 $$\begin{aligned} I_2&= \int _{\Omega }\left( \Delta ^2(u_2\partial _2f) - u_2\Delta ^2\partial _2f\right) \Delta ^2f dx \nonumber \\&= \int _{\Omega }\left( \Delta ^2(u_2\partial _2(f-\overline{f})) - u_2\Delta ^2\partial _2(f-\overline{f})\right) \Delta ^2f dx \nonumber \\&\quad + \int _{\Omega }\left( \Delta ^2(u_2\partial _2\overline{f}) - u_2\Delta ^2\partial _2\overline{f}\right) \Delta ^2{f} dx\nonumber \\&=: I_{21}+ I_{22}. \end{aligned}$$

Again, the Cauchy-Schwarz inequality gives us

$$ |I_{21}|\le \Vert \Delta ^2(u_2(f-\overline{f})) - u_2\Delta ^2\partial _2(f-\overline{f})\Vert _{L^2}\Vert \Delta ^2f\Vert _{L^2}, $$

while Lemma 3.4 yields

$$\begin{aligned} \Vert \Delta ^2(u_2(f-\overline{f})) - u_2\Delta ^2\partial _2(f-\overline{f})\Vert _{L^2}&\le _C \Vert u_2\Vert _{H^4}\Vert \partial _2(f-\overline{f})\Vert _{L^\infty } + \Vert u_2\Vert _{W^{1,\infty }}\Vert f-\overline{f}\Vert _{H^4}\\&\le _C \Vert u_2\Vert _{H^4}\Vert \partial _{12}f\Vert _{{H}^1} + \Vert u_2\Vert _{W^{1,\infty }}\Vert f\Vert _{H^4}\\&\le _C \Vert u_2\Vert _{H^4}\Vert \partial _{1}\Delta f\Vert _{L^2} + \Vert u_2\Vert _{W^{1,\infty }}\Vert \Delta ^2f\Vert _{L^2}. \end{aligned}$$

where the second inequality is due to $\Vert \overline{f}\Vert _{H^4(\Omega )}\le \Vert f\Vert _{H^4(\Omega )}$ and Lemma 3.5, and the last inequality follows from Lemma 4.3. Hence, we get

4.13 $$\begin{aligned} |I_{21}|\le \left( \Vert u_2\Vert _{H^4}\Vert \partial _{1}\Delta f\Vert _{L^2} + \Vert u_2\Vert _{W^{1,\infty }}\Vert \Delta ^2f\Vert _{L^2}\right) \Vert \Delta ^2 f\Vert _{L^2}. \end{aligned}$$

Next, we estimate $I_{22}$. Recalling $I_{22}$ from (4.12) and applying (4.11) with $g=u_2, \ h=\partial _2\overline{f}$, we have

4.14 $$\begin{aligned} I_{22}&= \int _{\Omega } \Delta ^2 u_2 \partial _2\overline{f}\Delta ^2f dx \nonumber \\&\quad + \int _{\Omega }(4\nabla \Delta u_2\cdot \nabla \partial _2\overline{f} +2\left( \nabla \partial _1u_2\cdot \nabla \partial _{12}\overline{f}\right. \nonumber \\&\quad \left. + \nabla \partial _2u_2\cdot \nabla \partial _{22}\overline{f}+\Delta u_2\Delta \partial _2\overline{f} + \nabla u_2\cdot \nabla \Delta \partial _2\overline{f}\right) )\Delta ^2f dx\nonumber \\&= \int _{\Omega } \Delta ^2 u_2 \partial _2\overline{f}\Delta ^2f dx +\int _{\Omega }(4\partial _2\Delta u_2\cdot \partial _{22}\overline{f} \nonumber \\&\quad +2\left( (\partial _{22}u_2+\Delta u_2)\cdot \partial _{222}\overline{f} + \partial _2 u_2\cdot \partial _{2222}\overline{f}\right) )\Delta ^2f dx\nonumber \\&=: I_{221}+ I_{222}, \end{aligned}$$

where the second last equality is due to the fact that $\overline{f}$ does not depend on the variable $x_1$. For $I_{222}$, using the Cauchy-Schwarz inequality, we just need a crude estimate,

4.15 $$\begin{aligned} |I_{222}|&\le _C \left( \Vert u_2\Vert _{H^3(\Omega )}\Vert \partial _{22}\overline{f}\Vert _{L^\infty }+ \Vert u_2\Vert _{W^{2,\infty }}\Vert f\Vert _{H^4}\right) \Vert \Delta ^2f\Vert _{L^2}\nonumber \\&\le _C \left( \Vert u_2\Vert _{H^3} + \Vert u_2\Vert _{W^{2,\infty }}\right) \Vert f\Vert _{H^4}\Vert \Delta ^2f\Vert _{L^2}\nonumber \\&\le _C \left( \Vert u_2\Vert _{H^3} + \Vert u_2\Vert _{W^{2,\infty }}\right) \Vert \Delta ^2f\Vert _{L^2}^2, \end{aligned}$$

where we used the Sobolev embedding $L^\infty ([0,1])\hookrightarrow H^1([0,1])$ to have $\Vert \partial _{22}\overline{f}\Vert _{L^\infty }\le \Vert {f}\Vert _{H^4}$ in the the second inequality, and we used Lemma 4.3 in the last inequality.

Lastly, we estimate $I_{221}$. Let $\Psi$ be the stream function of *u* such that

$$ \int _{\Omega }\Psi dx=0,\quad u=\nabla ^{\perp }\Psi \text { with }\Psi =\nabla \Psi =0 \text { on }\partial \Omega . $$

Indeed, such a stream function exists since *u* is divergence free and, especially, $u=0$ on $\partial \Omega$. Using the integration by parts, we get

$$\begin{aligned} I_{221}&=\int _{\Omega }\partial _1 \Delta ^2\Psi \partial _2\overline{f}\Delta ^2 f dx = -\int \left( \Delta ^2 \Psi \partial _2\overline{f}\right) \Delta ^2\partial _1f dx =-\int \Delta \left( \Delta ^2 \Psi \partial _2\overline{f}\right) \Delta \partial _1f dx, \end{aligned}$$

where the integration by parts in the last equality is justified by the assumption that $\partial _2\theta =\partial _{22}\overline{f}=0$ on $\partial \Omega$. Therefore, expanding $\Delta (\Delta ^2\Psi \partial _2\overline{f})$, we get

$$\begin{aligned} I_{221}&= -\int (\Delta ^3\Psi \partial _2\overline{f} + 2\partial _2\Delta ^2\Psi \cdot \partial _{22}\overline{f} + \Delta ^2\Psi \partial _{222}\overline{f})\partial _1\Delta \theta dx. \end{aligned}$$

Then, we obtain

$$\begin{aligned} |I_{221}|&\le \Vert \Psi \Vert _{H^6}\Vert \overline{f}\Vert _{W^{3,\infty }}\Vert \partial _1\Delta f\Vert _{L^2} \le \Vert u\Vert _{H^5}\Vert \partial _1\Delta f\Vert _{L^2}\Vert \overline{f}\Vert _{H^4}\le \Vert u\Vert _{H^5}\Vert \partial _1\Delta f\Vert _{L^2}\Vert \Delta ^2 f\Vert _{L^2}, \end{aligned}$$

where we used Lemma 4.3 in the last inequality. Combining this with (4.15) and (4.14), we get

$$ |I_{22}|\le \left( \Vert u_2\Vert _{H^3} + \Vert u_2\Vert _{W^{2,\infty }}\right) \Vert \Delta ^2f\Vert _{L^2}^2 +\Vert u\Vert _{H^5}\Vert \partial _1\Delta f\Vert _{L^2}\Vert \Delta ^2 f\Vert _{L^2}. $$

Combining this with (4.13) and (4.12), we have

$$ |I_2|\le \left( \Vert u_2\Vert _{H^3} + \Vert u_2\Vert _{W^{2,\infty }}\right) \Vert \Delta ^2f\Vert _{L^2}^2 +\Vert u\Vert _{H^5}\Vert \partial _1\Delta f\Vert _{L^2}\Vert \Delta ^2 f\Vert _{L^2}. $$

Combining this with (4.10) and (4.9), we get

$$ \left| \int _{\Omega } \Delta ^2(u\cdot \nabla \theta )\Delta ^2\theta dx \right| \le \left( \Vert u_2\Vert _{H^3} + \Vert u_2\Vert _{W^{2,\infty }}\right) \Vert \Delta ^2f\Vert _{L^2}^2 +\Vert u\Vert _{H^5}\Vert \partial _1\Delta f\Vert _{L^2}\Vert \Delta ^2 f\Vert _{L^2}. $$

This finishes the proof.

### Analysis of the energy structure

As in the previous section for the IPM equation, we define

4.16 $$\begin{aligned} E(t):= \int _{\Omega }(\rho (t)-\rho _0^*)x_2dx,\quad K(t):=\Vert \Delta \Psi (t)\Vert _{L^2}^2, \end{aligned}$$

where $\rho _0^*$ is the vertical rearrangement of the initial density $\rho _0$. Throughout this subsection, we will assume that

4.17 $$\begin{aligned} \Vert \theta (t)\Vert _{H^3}^2+ \int _0^T \Vert \Delta ^2 \Psi (t)\Vert _{H^2}^2 dt\le \delta \le \delta _0\ll 1, \text { for } t\in [0,T]\hbox { for some }T>0\hbox { and }\delta _0>0. \end{aligned}$$

#### Proposition 4.6

There exists $\delta _0=\delta _0(\gamma ,\Vert \rho _s\Vert _{H^4})>0$ such that if (4.17) holds, then

4.18 $$\begin{aligned} \frac{d}{dt}E(t)= -K(t),\quad \frac{d}{dt}K(t)\le -C \Vert u_2\Vert _{L^2}^2. \end{aligned}$$

#### Proof

Differentiating the energy difference *E*(*t*) in time, we see that

4.19 $$\begin{aligned} \frac{d}{dt}E(t) = \int _{\Omega }\rho _t x_2dx = -\int _{\Omega }u\cdot \nabla \rho x_2dx = \int _{\Omega }u_2\rho dx. \end{aligned}$$

Using (1.5), we can further simplify the last expression as

$$ \int _{\Omega }u_2\rho dx = \int _{\Omega }\partial _1\Psi \rho dx = -\int _{\Omega }\Psi \partial _1\rho dx =- \int _{\Omega }\Psi \Delta ^2 \Psi dx = -\int _{\Omega }|\Delta \Psi |^2 dx, $$

therefore, we get

4.20 $$\begin{aligned} \frac{d}{dt}E(t) =-\int _{\Omega }|\Delta \Psi |^2 dx = -K(t). \end{aligned}$$

In order to estimate $\frac{d}{dt}K(t)$, taking a derivative in (4.19), we compute

$$ \frac{1}{2}\left( \frac{d}{dt}\right) ^2 E(t) =\frac{1}{2} \frac{d}{dt}\int _{\Omega } u_2\rho dx = \frac{1}{2}\int _{\Omega } u_2 \rho _t dx + \frac{1}{2}\int _{\Omega }\partial _tu_2 \rho dx. $$

Using $u_2=\partial _1\Psi$ and $\partial _1\rho =\Delta ^2\Psi$, we have

$$\begin{aligned} \int _{\Omega }\partial _tu_2 \rho dx&= -\int _{\Omega }\partial _t\Psi \partial _1\rho dx =- \int _{\Omega }\partial _t\Psi \Delta ^2 \Psi dx=- \int _{\Omega }\partial _t\Delta ^2 \Psi \Psi dx \\  &= -\int _{\Omega }{\partial _t}\partial _1\rho \Psi dx = \int _{\Omega }\partial _t\rho u_2 dx \end{aligned}$$

Therefore, we get

$$\begin{aligned} \frac{1}{2}\left( \frac{d}{dt}\right) ^2 E(t)&= \int _{\Omega }u_2\rho _t = -\int _{\Omega } u_2 u \cdot \nabla \rho dx = -\int _{\Omega }u_2^2\partial _2\rho dx - \int _{\Omega }u_2u_1\partial _1\rho dx\\&\ge -\int _{\Omega }u_2^2\partial _2\rho dx - \Vert u_2\Vert _{L^2}\Vert u_1\partial _1\rho \Vert _{L^2}. \end{aligned}$$

Using the assumption on $\Vert \theta \Vert _{H^3}$ in (4.17), we have

$$ -\int _{\Omega }u_2^2\partial _2\rho dx = -\int u_2^2 \partial _2\rho _s dx - \int u_2^2 \partial _2(\rho -\rho _s)dx\ge \gamma \Vert u_2\Vert _{L^2}^2 -\sqrt{\delta _0}\Vert u_2\Vert _{L^2}^2\ge C\Vert u_2\Vert _{L^2}^2, $$

where we used the strict positivity of $\gamma$ which is assumed in (4.1). Using (4.20), we see that the above inequality implies

4.21 $$\begin{aligned} \frac{d}{dt}K(t) + C\Vert u_2\Vert _{L^2}^2 \le C\Vert u_2\Vert _{L^2}\Vert u_1\partial _1\rho \Vert _{L^2}. \end{aligned}$$

Let us estimate $\Vert u_1\partial _1\rho \Vert _{L^2}^2$. Using (1.5), we rewrite

4.22 $$\begin{aligned} \Vert u_1\partial _1\rho \Vert _{L^2(\Omega )} = \Vert \partial _2\Psi \Delta ^2\Psi \Vert _{L^2}\le \Vert \nabla \Psi \Vert _{L^4}\Vert \Delta ^2\Psi \Vert _{L^4} \le \Vert \Delta ^2\Psi \Vert _{H^2}\Vert \Psi \Vert _{L^2}, \end{aligned}$$

where the last inequality is due to the Gagliardo-Nirenberg interpolation theorem. Moreover, we notice from (1.5) that $g(x_2):=\int _{\mathbb {T}}\Psi (x_1,x_2)dx_1$ satisfies

$$\begin{aligned} \partial _{2222}g(x_2)&= \int _{\mathbb {T}}\partial _{2222}\Psi (x_1,x_2)dx_1 = \int _{\mathbb {T}}\Delta ^2 \Psi (x_1,x_2) - (\partial _{1111}+2\partial _{1122})\Psi (x_1,x_2)dx_1\\  &=\int _{\mathbb {T}}\partial _1\rho dx=0, \end{aligned}$$

with the boundary condition, $g=\partial _2 g=0$ on $\partial \Omega$. Therefore, $g=0$ for all $x_2\in [0,1]$. In other words, the map $x_1\rightarrow \Psi (x_1,x_2)$ has zero average for each fixed $x_2$. Then, the Poincaré inequality tells us

$$ \Vert \Psi \Vert _{L^2}\le \Vert \partial _1\Psi \Vert _{L^2}=\Vert u_2\Vert _{L^2}. $$

Substituting this into (4.22), we get

$$ \Vert u_1\partial _1\rho \Vert _{L^2}\le \Vert \Delta ^2\Psi \Vert _{H^2}\Vert u_2\Vert _{L^2}\le \Vert \rho -{\rho }_s\Vert _{H^3}\Vert u_2\Vert _{L^2}\le \sqrt{\delta _0} \Vert u_2\Vert _{L^2}, $$

where the second inequality follows from Lemma 4.1 (noting that $\Delta ^2\Psi = \partial _1(\rho -\rho _s)$ and $\rho -\rho _s=0$ on $\partial \Omega$) and the last inequality follows from (4.17). Plugging this into (4.21), we obtain that for sufficiently small $\delta >0$, $\frac{d}{dt}K(t)\le - C\Vert u_2\Vert _{L^2}^2.$ Combining this with (4.20), we finish the proof of the proposition.

#### Corollary 4.7

There exists $\delta _0=\delta _0(\gamma ,\Vert \rho _s\Vert _{H^4})>0$ such that if (4.17) holds, then,

$$\begin{aligned} E(t)&\le \frac{C\delta }{t^2},\\ \frac{2}{t}\int _{t/2}^{t} K(s)ds&\le \frac{C\delta }{t^{3}}, \\ \frac{2}{t}\int _{t/2}^{t}\Vert u_2(s)\Vert _{L^2}^2 ds&\le \frac{C\delta }{t^{4}}, \end{aligned}$$

for all $$t\in [0,T]$$.

#### Proof

Thanks to Proposition 2.5, we know that there is a constant $C>0$ such that

4.23 $$\begin{aligned} C^{-1}\Vert \rho (t)-\rho _0^*\Vert _{L^2}^2\le E(t)\le C\Vert \rho (t)-\rho _0^*\Vert _{L^2}^2 . \end{aligned}$$

Now, using the Gagliardo-Nirenberg interpolation theorem, we estimate

$$ \Vert \partial _1\rho \Vert _{L^2(\Omega )} =\Vert \Delta ^2\Psi \Vert _{L^2}\le C \Vert \Delta ^2\Psi \Vert _{H^2}^{1/2}\Vert \Delta \Psi \Vert _{L^2(\Omega )}^{1/2}. $$

On the other hand, applying (2.28), we observe that $\Vert \partial _1\rho \Vert _{L^2}\ge C\Vert \rho -\rho _0^*\Vert _{L^2}$. Combining this with the above estimate, we get

$$ \Vert \Delta \Psi \Vert _{L^2}^2\ge \Vert \Delta ^2\Psi \Vert _{H^2}^{-2}\Vert \rho -\rho _0^*\Vert _{L^2}^4\ge C \Vert \Delta ^2 \Psi \Vert _{H^2}^{-2} E(t)^2. $$

where the last inequality follows from (4.23). Hence, the variation of the potential energy in (4.18) yields

4.24 $$\begin{aligned} \frac{d}{dt}E(t)\le - C\left( \Vert \Delta ^2 \Psi \Vert _{H^2}^2\right) ^{-1} E(t)^{2}. \end{aligned}$$

Applying Lemma 2.1 with $\alpha =1>0$ and $n=2$ and $a(t)=\Vert \Delta ^2\Psi \Vert _{H^2}^2$, we get

$$ E(t)\le \frac{CA}{t^{2}}, \text { where }A=\int _0^t \Vert \Delta ^2 \Psi \Vert _{H^2}^{2}ds. $$

Under the assumptions in (3.30), we have $A\le C\delta$. Therefore,

4.25 $$\begin{aligned} E(t)\le \frac{C\delta }{t^{2}}. \end{aligned}$$

Applying Lemma 2.2 with $f(t)=E(t),\ g(t)=K(t),\ h(t)=C\Vert u_2(t)\Vert _{L^2}^2$, it follows from (4.18) that

$$ \frac{2}{t}\int _{t/2}^{t} K(s)ds\le \frac{C}{t^{3}},\text { and } \frac{2}{t}\int _{t/2}^{t}\Vert u_2(s)\Vert _{L^2}^2 ds \le \frac{C\delta }{t^{4}}. $$

Together with (4.25), we obtain the desired estimates.

### Proof of Theorem 1.6

Let $\delta _0$ be fixed so that Proposition 4.6 and Corollary 4.7 are applicable. We claim that there exists $\epsilon =\epsilon (\gamma ,\Vert \Vert \rho _s\Vert _{H^4})>0$ such that if $\rho _0-{\rho }_s\in H^2_0(\Omega )\cap H^4(\Omega )$ and

4.26 $$\begin{aligned} \Vert \rho _0-{\rho }_s\Vert _{H^4}\le \epsilon \ll 1,\end{aligned}$$

then for all $t>0$, it holds that

4.27 $$\begin{aligned} \Vert \Delta ^2 \theta (t)\Vert _{L^2(\Omega )}^2 +\int _0^t \Vert \Delta ^2 \Psi (t)\Vert _{H^2(\Omega )}^2 dt< C\epsilon ^2,\text { for some }C>0. \end{aligned}$$

Let us suppose for the moment that the claim is true. Then by Corollary 4.7, the solution $\rho (t)$ satisfies

4.28 $$\begin{aligned} E(t)\le \frac{C\epsilon ^2}{t^2},\quad \frac{2}{t}\int _{t/2}^{t} K(s)ds\le \frac{C\epsilon ^2}{t^{3}}, \quad \frac{2}{t}\int _{t/2}^{t}\Vert u_2(s)\Vert _{L^2}^2 ds \le \frac{C\epsilon ^2}{t^{4}},\text { for all }t>0. \end{aligned}$$

Especially, the potential energy estimate in (4.28) and (2.27) give us all the necessary properties to establish Theorem 1.6.

In the rest of the proof, we aim to prove (4.27). Towards a contradiction, let $T^*>0$ be the first time that (4.27) breaks down, that is,

4.29 $$\begin{aligned} \Vert \Delta ^2 \theta (T^*)\Vert _{L^2(\Omega )}^2 +\int _0^{T^*} \Vert \Delta ^2 \Psi (t)\Vert _{H^2(\Omega )}^2 dt= M\epsilon ^2\ll 1 , \end{aligned}$$

for some $M\gg 1$, which will be chosen later. Towards a contradiction, let us denote

$$ f(t):= \Vert \Delta ^2\theta (t) \Vert _{L^2}^2,\quad g(t):=\Vert \Delta ^2\Psi (t)\Vert _{H^2}^2.$$

Since $$\theta _0:=\rho _0-\overline{\rho }\in H^2_0\cap H^4$$, it follows from Theorem 4.2 that $$\theta (t)$$ satisfies $$\theta =\partial _2\theta =\partial _{22}\overline{\theta }=0$$ on $$\partial \Omega $$. Then, Lemma 4.3 and our assumption on $$T^* $$ tell us that

4.30 $$\begin{aligned} C^{-1}g(t)&\le {C}^{-1}\Vert \theta (t)\Vert _{H^4}^2\le f(t)\le C\Vert \theta (t)\Vert _{H^4}^2 \nonumber \\  &\le CM\epsilon ^2, \text { for all } t\in [0,T^*],\quad f(0)\le C\epsilon ^2, \end{aligned}$$

where the initial condition is due to (4.26). In particular, we have

$$ \Vert \partial _1\Delta \theta \Vert _{L^2}^2=\Vert \Delta \partial _1\theta \Vert _{L^2}^2 \ge \Vert \partial _1\theta \Vert _{H^2}^2 = \Vert \Delta ^2\Psi \Vert _{H^2}^2 = g(t). $$

Using this and (4.30), we derive from Proposition 4.4 that

4.31 $$\begin{aligned} \frac{d}{dt}f(t)&\le -Cg(t) + C\left( \Vert u_2\Vert _{H^3} + \Vert u_2\Vert _{W^{2,\infty }}\right) f(t) \nonumber \\  &\quad + C \Vert u_2\Vert _{H^3}\Vert \Delta ^2\theta \Vert _{L^2}+\Vert \partial _1\theta \Vert _{L^2}^2,\text { for }t\in [0,T^*]. \end{aligned}$$

With this differential inequality, we first derive a crude estimate for "short time" first and more careful analysis will follows afterwards.

Using the Sobolev embedding $W^{2,\infty }(\Omega )\hookrightarrow H^4(\Omega )$, we have

4.32 $$\begin{aligned} \Vert u_2\Vert _{H^3} + \Vert u_2\Vert _{W^{2,\infty }}\le \Vert u_2\Vert _{H^4} \le \Vert \nabla \Psi \Vert _{H^4} \le \Vert \partial _1\theta \Vert _{H^1}\le \sqrt{f(t)}, \end{aligned}$$

where the last inequality follows from (4.30). From this and (4.31) we see that *f* satisfies

$$ \frac{d}{dt}f(t) \le C(f(t)^{3/2} + f(t))\le C f(t),\quad \text { for } t\in [0,T^*]. $$

Using $f(0)\le C\epsilon ^2$, we can immediately deduce from this differential inequality that $f(t)\le C\epsilon ^2e^{Ct},$ for $t\in [0,T^*]$. Since $g(t)\le Cf(t)$, we can easily deduce that

4.33 $$\begin{aligned} f(t) + \int _0^{t}g(s)ds\le C\epsilon ^2 e^{Ct}\text { for }t\in [0,T^*]. \end{aligned}$$

Thus, for (4.29) to occur, we must have $M\epsilon ^2 \le _C \epsilon ^2 e^{CT^*}$. This gives us a lower bound of $T^*$,

$$ T^*\ge C \log M. $$

Let us pick

4.34 $$\begin{aligned} T^*_M:=\log \log M\gg 1. \end{aligned}$$

Without loss of generality, we can assume *M* is sufficiently large to ensure that

$$ T^*_M < T^*. $$

Then , it follows from (4.33) that

4.35 $$\begin{aligned} f(T^*_M) + \int _0^{T^*_M} g(t)dt \le C\epsilon ^2 (\log M)^C. \end{aligned}$$

Now, we will estimate *f*(*t*) more carefully for $t \ge T^*_M$. Using the Gagliardo-Nirenberg interpolation theorem, we see that

4.36 $$\begin{aligned} \Vert u_2\Vert _{W^{2,\infty }} +\Vert u_2\Vert _{H^3}\le _C \Vert u_2\Vert _{L^2}^{2/5}\Vert u_2\Vert _{H^5}^{3/5}\le _C \Vert u_2\Vert _{L^2}^{2/5}\Vert \Delta ^2 \Psi \Vert _{H^2}^{3/5}. \end{aligned}$$

Hence, Young’s inequality can give us

4.37 $$\begin{aligned} \left( \Vert u_2\Vert _{W^{2,\infty }} +\Vert u_2\Vert _{H^3} \right) f(t)&\le _C \Vert u_2\Vert _{L^2}^{2/5}\Vert \Delta ^2 \Psi \Vert _{H^2}^{3/5}f(t) \nonumber \\&\le _C \eta \Vert \Delta ^2\Psi \Vert _{H^2}^2 + C_\eta f(t)^{10/7}\Vert u_2\Vert _{L^2}^{4/7}\nonumber \\&\le _C \eta g(t) + C_\eta (M\epsilon ^2)^{10/7}\Vert u_2\Vert _{L^2}^{4/7}, \text { for any }\eta >0. \end{aligned}$$

Again using (4.36) and the estimate $\Vert \theta (t)\Vert _{H^4}\le C\sqrt{M\epsilon ^2}$ in (4.30), we get

4.38 $$\begin{aligned} \Vert u_2\Vert _{H^3}\Vert \Delta ^2\theta \Vert _{L^2}&\le _C\Vert u_2\Vert _{L^2}^{2/5}\Vert \Delta ^2\Psi \Vert _{H^2}^{3/5}\sqrt{M\epsilon ^2}\nonumber \\&= \Vert u_2\Vert _{L^2}^{2/5}\sqrt{M\epsilon ^2}g(t)^{3/10}\nonumber \\&\le _C \eta g(t)+ C_\eta \left( \sqrt{M\epsilon ^2}\right) ^{10/7}\Vert u_2\Vert _{L^2}^{4/7},\text { for any }\eta >0, \end{aligned}$$

where the last inequality follows from Young’s inequality. Similarly, the Gagliardo-Nirenberg interpolation theorem and the definition of *K*(*t*) in (4.16) give us

4.39 $$\begin{aligned} \Vert \partial _1\theta \Vert _{L^2}^2&=\Vert \Delta ^2\Psi \Vert _{L^2}^2\le C\Vert \Delta ^2\Psi \Vert _{H^2}\Vert \Delta \Psi \Vert _{L^2}\le \eta \Vert \Delta ^2\Psi \Vert _{H^2}^2 \nonumber \\  &\quad + C_\eta \Vert \Delta \Psi \Vert _{L^2}^2\le _C \eta g(t) + C_\eta K(t). \end{aligned}$$

Summing up this, (4.38) and (4.37), we obtain

$$\begin{aligned} \left( \Vert u_2\Vert _{W^{2,\infty }} +\Vert u_2\Vert _{H^3} \right) f(t)&+ \Vert u_2\Vert _{H^3}\Vert \Delta ^2\theta \Vert _{L^2} + \Vert \partial _1\theta \Vert _{L^2}^2\\&\le C\eta g(t)+C_\eta (\sqrt{M\epsilon ^2})^{10/7}\Vert u_2\Vert _{L^2}^{4/7} + K(t). \end{aligned}$$

Substituting this into (4.31) and choosing $\eta$ small enough, we arrive at

4.40 $$\begin{aligned} \frac{d}{dt}f(t) +Cg(t)\le _C \left( \sqrt{M\epsilon ^2}\right) ^{10/7}\Vert u_2\Vert _{L^2}^{4/7}+K(t),\text { for }t\in [0,T^*]. \end{aligned}$$

The estimate for $\Vert u_2\Vert _{L^2}$ in Corollary 4.7 tells us that

$$ \frac{2}{t}\int _{t/2}^{t} s^{1/4}\Vert u_2(s)\Vert _{L^2}^2ds\le C t^{1/4}\frac{2}{t}\int _{t/2}^t\Vert u_2(s)\Vert _{L^2}^2 ds\le C\frac{M\epsilon ^2}{t^{15/4}},\text { for all }t\in [0,T^*]. $$

Hence applying Lemma 2.3 with $\alpha = 2/7$, $n=15/4$, we obtain

$$ \int _1^{T^*} t^{1/14}\Vert u_2(t)\Vert _{L^2}^{4/7}dt= \int _1^{T^*} \left( t^{1/4}\Vert u_2(t)\Vert _{L^2}^2\right) ^{2/7}dt\le C \left( M\epsilon ^2\right) ^{2/7}. $$

Thanks to (4.34), this implies

4.41 $$\begin{aligned} \int _{T_M^*}^{T^*}\left( \sqrt{M\epsilon ^2}\right) ^{10/7}\Vert u_2(t)\Vert _{L^2}^{4/7}dt&\le \left( \sqrt{M\epsilon ^2}\right) ^{10/7} (T_M^*)^{-1/14}\int _{T^*_M}^{T^*}t^{1/14}\Vert u_2(t)\Vert _{L^2}^{4/7}dt \nonumber \\  &\le CM\epsilon ^2(T^*_M)^{-1/14}. \end{aligned}$$

Similarly, for the term *K*(*t*) in (4.40), we use Corollary 4.7 to see

$$ \frac{2}{t}\int _{t/2}^{t}s^{1/14}K(s)ds \le C t^{1/14}\frac{2}{t}\int _{t/2}^{t} K(s)ds\le C\frac{M\epsilon ^2}{t^{41/14}}. $$

Applying Lemma 2.3 with $\alpha =1$ and $n=41/14$, we get $\int _1^{T^*}t^{1/14}K(t)dt \le CM\epsilon ^2$. Therefore

$$ \int _{T^*_M}^{T^*}K(s)ds\le \left( T^*_M\right) ^{-1/14}\int _{T^*_M}^{T^*}s^{1/14} K(s)ds\le C(T^*_M)^{-1/14}M\epsilon ^2. $$

Combining this with (4.41), integrating (4.40) over $t\in [T_M^*,T^*]$ gives

$$ f(T^*) + \int _{T_M^*}^{T^*} g(s) ds\le _C f(T^*_M) + \left( T^*_M\right) ^{-1/14} M\epsilon ^2 $$

Together with (4.35) and (4.34), this estimate finally gives us

$$ f(T^*)+\int _{0}^{T^*}g(t)dt\le _CM\epsilon ^2\left( M^{-1}(\log M)^C + (\log \log M)^{-1/14} \right) . $$

Comparing this to (4.29), we must have

$$ 1\le C\left( M^{-1}(\log M)^C + (\log \log M)^{-1/14} \right) , $$

which leads to a contradiction if *M* is chosen sufficiently large depending only on the implicit constant *C*. This finishes the proof.

## Data Availability

Data sharing not applicable to this article as no datasets were generated or analysed during the current study.

## Proof of Lemma 3.1

We argue by induction on *k*, that is, we will prove that for every $k\in \mathbb {N}$,

5.1 $$\begin{aligned} \Vert \partial _{1}^n\partial _2^kf\Vert _{L^2}\le C_{n,k}\left( \Vert \partial _1^{n+k}f\Vert _{L^2} +\Vert \partial _2^{n+k}f\Vert _{L^2} \right) ,\text { for all } n\in \mathbb {N}\cup \left\{ 0\right\} \hbox { and }f\in X^\infty . \end{aligned}$$

When $k=0$, the above estimate holds true trivially. Now, let us aim to prove (5.1) for $k\rightarrow k+1$, assuming that the statement is true for some $k\ge 0$. We split the cases $n=0, 1$ and $n\ge 2$.

**Case: **$n=0$. It is trivial that

5.2 $$\begin{aligned} \Vert \partial _2^{k+1}f\Vert _{L^2}\le C_{k}\left( \Vert \partial _1^{k+1}f\Vert _{L^2} +\Vert \partial _2^{k+1}f\Vert _{L^2} \right) . \end{aligned}$$

**Case: **$n=1$. We have

$$\begin{aligned} \Vert \partial _1 \partial _2^{k+1}f\Vert _{L^2}^2&=\int _\Omega \partial _1\partial _2^{k+1}f(x)\partial _1\partial _2^{k+1}f(x)dx = -\int _{\Omega }\partial _{11}\partial _2^{k+1}f(x)\partial _2^{k+1}f(x)dx \\&= \int _{\Omega } \partial _{11}\partial _2^{k}f(x)\partial _{2}^{k+2}f(x)dx\le \Vert \partial _{11}\partial _2^kf\Vert _{L^2}^2 + \Vert \partial _2^{k+2}f\Vert _{L^2}^2, \end{aligned}$$

where the integration by parts to get the second equality is justified by (3.5). Using the induction hypothesis (5.1) for *k*, we have $\Vert \partial _{11}\partial _2^kf\Vert _{L^2}^2\le C_k\left( \Vert \partial _1^{k+2}f\Vert _{L^2}^2 +\Vert \partial _2^{k+2}f\Vert _{L^2}^2 \right)$, hence

5.3 $$\begin{aligned} \Vert \partial _1 \partial _2^{k+1}f\Vert _{L^2}^2 \le C_k\left( \Vert \partial _1^{k+2}f\Vert _{L^2}^2 +\Vert \partial _2^{k+2}f\Vert _{L^2}^2 \right) . \end{aligned}$$

**Case: **$n\ge 2$. We claim that

5.4 $$\begin{aligned} \Vert \partial _{1}^{n-j}\partial _2^{k+1+j}f\Vert ^2_{L^2(\Omega )}&\le \frac{1}{2}\left( \Vert \partial _1^{n-j+1}\partial _2^{k+j}f\Vert _{L^2}^2 + \Vert \partial _1^{n-j-1}\partial _2^{k+j+2}f\Vert _{L^2}^2\right) ,\nonumber \\&\text { for all }0\le j\le n-1. \end{aligned}$$

Suppose (5.4) holds true for the moment. Then we have

5.5 $$\begin{aligned} \sum _{j=0}^{n-1} \Vert \partial _{1}^{n-j}\partial _2^{k+1+j}f\Vert ^2_{L^2(\Omega )}&\le \frac{1}{2} \sum _{j=0}^{n-1}\Vert \partial _1^{n-j+1}\partial _2^{k+j}f\Vert _{L^2}^2 \nonumber \\  &\quad +\frac{1}{2} \sum _{j=0}^{n-1} \Vert \partial _1^{n-j-1}\partial _2^{k+j+2}f\Vert _{L^2}^2=:S_1+S_2 \end{aligned}$$

The first summation in the right-hand side can be written as

$$\begin{aligned} S_1&= \frac{1}{2}\left( \Vert \partial _1^{n+1}\partial _2^{k}f\Vert _{L^2}^2 + \sum _{j=0}^{n-2}\Vert \partial _1^{n-j}\partial _2^{k+j+1}f\Vert _{L^2}^2\right) \\&= \frac{1}{2}\left( \Vert \partial _1^{n+1}\partial _2^{k}f\Vert _{L^2}^2 +\Vert \partial _1^n\partial _2^{k+1}f\Vert _{L^2}^2+ \sum _{j=1}^{n-2}\Vert \partial _1^{n-j}\partial _2^{k+j+1}f\Vert _{L^2}^2\right) \end{aligned}$$

and the second summation can be written as

$$\begin{aligned} S_2&=\frac{1}{2}\left( \sum _{j=1}^{n-1}\Vert \partial _1^{n-j}\partial _2^{k+j+1}f\Vert _{L^2}^2 + \Vert \partial _2^{n+k+1}f\Vert _{L^2}^2\right) \\&=\frac{1}{2}\left( \sum _{j=1}^{n-2}\Vert \partial _1^{n-j}\partial _2^{k+j+1}f\Vert _{L^2}^2 + \Vert \partial _1^{1}\partial _2^{k+n}f\Vert _{L^2}^2 + \Vert \partial _2^{n+k+1}f\Vert _{L^2}^2 \right) \end{aligned}$$

Summing them up, we can write the right-hand side of (5.5) as

$$\begin{aligned} S_1+S_2&= \sum _{j=1}^{n-2} \Vert \partial _{1}^{n-j}\partial _2^{k+1+j}f\Vert ^2_{L^2(\Omega )} \\  &\quad +\frac{1}{2}\left( \Vert \partial _1^{n+1}\partial _2^{k}f\Vert _{L^2}^2 +\Vert \partial _1^n\partial _2^{k+1}f\Vert _{L^2}^2 + \Vert \partial _1\partial _2^{k+n}f\Vert _{L^2}^2 + \Vert \partial _2^{n+k+1}f\Vert _{L^2}^2 \right) \end{aligned}$$

Plugging this into (5.5), and after the cancellations of the summation in *j* over 1 to $n-2$, we obtain

$$\begin{aligned} \Vert \partial _1^n\partial _2^{k+1}f\Vert _{L^2}^2&+\Vert \partial _1\partial _2^{k+n}f\Vert _{L^2}^2\\  &\le \frac{1}{2}\left( \Vert \partial _1^{n+1}\partial _2^{k}f\Vert _{L^2}^2 +\Vert \partial _1^n\partial _2^{k+1}f\Vert _{L^2}^2 + \Vert \partial _1\partial _2^{k+n}f\Vert _{L^2}^2 + \Vert \partial _2^{n+k+1}f\Vert _{L^2}^2 \right) , \end{aligned}$$

which is equivalent to

$$ \Vert \partial _1^n\partial _2^{k+1}f\Vert _{L^2}^2+\Vert \partial _1\partial _2^{k+n}f\Vert _{L^2}^2 \le \Vert \partial _1^{n+1}\partial _2^{k}f\Vert _{L^2}^2 + \Vert \partial _2^{n+k+1}f\Vert _{L^2}^2. $$

Hence, dropping the second term in the left-hand side, we get

$$ \Vert \partial _1^n\partial _2^{k+1}f\Vert _{L^2}^2\le \Vert \partial _1^{n+1}\partial _2^{k}f\Vert _{L^2}^2 + \Vert \partial _2^{n+k+1}f\Vert _{L^2}^2\le C_{n,k}\left( \Vert \partial _1^{n+k+1}f\Vert _{L^2}^2+ \Vert \partial _2^{n+k+1}f\Vert _{L^2}^2\right) , $$

where we used the induction hypothesis (5.1) for *k*. Combining this with (5.2) and (5.3), we see that the statement (5.1) holds true for all *n*, for $k+1$. The only missing part is a proof of (5.4).

**Proof of **(5.4). It is straightforward that

$$\begin{aligned} \Vert \partial _{1}^{n-j}\partial _2^{k+1+j}f\Vert ^2_{L^2(\Omega )}&= \int _{\Omega } \partial _{1}^{n-j}\partial _2^{k+1+j}f(x) \partial _{1}^{n-j}\partial _2^{k+1+j}f(x)dx\\&=-\int _{\Omega } \partial _1\partial _{1}^{n-j}\partial _2^{k+1+j}f(x) \partial _{1}^{n-j-1}\partial _2^{k+1+j}f(x)dx\\&= \int _{\Omega } \partial _{1}^{n-j+1}\partial _2^{k+j}f(x) \partial _{1}^{n-j-1}\partial _2^{k+2+j}f(x)dx\\&\le \frac{1}{2}\Vert \partial _1^{n-j+1}\partial _2^{k+j}f\Vert _{L^2}^2 + \frac{1}{2}\Vert \partial _1^{n-j-1}\partial _2^{k+2+j}f\Vert _{L^2}^2, \end{aligned}$$

where the second and the third equality follow from the integration by parts which is valid due to (3.5) and the last inequality follows from Young’s inequality. This proves the claim (5.4).
